# Structural covariance network alterations across the spectrum of cognitive status in Parkinson's disease

**DOI:** 10.1016/j.prdoa.2026.100456

**Published:** 2026-05-26

**Authors:** Haruto Shibata, Yuto Uchida, Keita Sakurai, Ken Tanaka, Yuya Kano, Noriyuki Matsukawa

**Affiliations:** a Department of Neurology, Nagoya City University East Medical Center, 1-2-23 Wakamizu, Chikusa-ku, Nagoya 464-8547, Japan; b Department of Neurology, Nagoya City University Graduate School of Medical Sciences, 1 Kawasumi, Mizuho-cho, Mizuho-ku, Nagoya 467-8601, Japan; c Department of Radiology and Radiological Science, Johns Hopkins University School of Medicine, 330 Traylor Building, 720 Rutland Avenue, Baltimore, MD 21205, USA; d Department of Radiology, NHO Higashinagoya National Hospital, 5-101, Umemorizaka, Meito-ku, Nagoya 465-8620, Japan; e Center for Frailty and Locomotive Syndrome, National Center for Geriatrics and Gerontology, Obu, Japan; f Department of Public Health, University of Hawaii at Manoa, Honolulu, USA.; g Department of Neurology, Toyokawa City Hospital, 23 Noji, Yawata-Cho, Toyokawa, Aichi 442-8561, Japan

**Keywords:** Parkinson's disease, Structural covariance network, Mild cognitive impairment

## Abstract

**Background:**

Parkinson's disease (PD) is a neurodegenerative disorder in which cognitive impairment frequently emerges as the disease progresses. Structural covariance network (SCN) analysis provides a framework for characterizing large-scale brain network organization beyond regional atrophy. This study aimed to assess alterations in SCN topology across the cognitive spectrum of PD.

**Methods:**

We retrospectively reviewed 16 healthy controls (HC), 20 patients with PD and normal cognition (PD-CN), and 22 patients with PD and mild cognitive impairment (PD-MCI). Cortical thickness–based SCNs were constructed, and graph-theoretical analyses were performed to assess global network metrics, nodal centrality, and modular organization.

**Results:**

Using a bootstrap-based area-under-the-curve framework (*N* = 1000 iterations per group; sparsity 0.15–0.40), a progressive increase in modularity was observed across HC, PD-CN, and PD-MCI (all Bonferroni-corrected *p* < 0.001), and global efficiency was selectively reduced in PD-MCI. Nodal analyses revealed widespread alterations in betweenness, closeness, and eigenvector centrality, with comparable spatial extents across all three metrics. Community detection using the Louvain algorithm showed progressive reorganization of modular structure, with less distinct intermodular boundaries in PD-CN and more spatially extended modules in PD-MCI.

**Conclusions:**

SCN analysis revealed stage-dependent network reorganization in PD, suggesting the utility of network-based approaches for characterizing large-scale brain alterations related to cognitive impairment.

## Highlights

• Cortical thickness–based structural covariance networks were constructed
• Graph-theoretical metrics quantified integration and segregation changes
• Progressive network disruption was observed from HC to PD-MCI
• Community reorganization revealed stage-dependent modular alterations
• Cognitive decline was associated with large-scale structural network changes

## 1 Introduction

Parkinson's disease (PD) is a common neurodegenerative disorder primarily characterized by motor symptoms. However, cognitive impairment is also commonly observed and constitutes a serious clinical issue, as it substantially compromises activities of daily living and overall quality of life [1]. Cognitive impairment in PD results from multifactorial pathological processes [2], including not only co-existing Alzheimer's pathology but also the propagation of α-synuclein into limbic and neocortical regions, corresponding to the advanced Braak stages of PD [3]. In addition, degeneration of the basal forebrain cholinergic system has been identified as a key contributor to cortical dysfunction associated with cognitive decline [2]. These mechanisms disrupt brain networks and contribute to cognitive impairment in PD [4].

Structural MRI studies have shown that cortical atrophy is associated with cognitive impairment in PD. Hippocampal atrophy has been reported as a key biomarker of cognitive decline in PD, particularly during the transitional stage to Parkinson's disease dementia (PDD) [5]. In Parkinson's disease with mild cognitive impairment (PD-MCI), cortical thinning has been consistently observed in the cingulate gyrus and parietal lobe [6]. In addition, cortical thinning of the insula has been identified in the early stages of PD with cognitive impairment and has been reported as a predictor of progression to dementia [5]. However, structural MRI studies have reported atrophy in different brain regions, and the reported patterns vary across studies. Resting-state functional MRI studies have shown that cognitive impairment in PD is associated with disruptions in large-scale networks, including the default mode, frontoparietal executive, attention, and salience networks [7], [8]. Graph-theoretical analyses have further suggested reduced network efficiency in PD-MCI [9], supporting the view that cognitive decline in PD involves network-level dysfunction.

Structural covariance network (SCN) analysis is a method for evaluating brain network organization using structural MRI measures such as cortical thickness or gray matter volume [6]. Conventional structural MRI approaches, such as voxel-based morphometry and surface-based morphometry, which assess cortical volume or thickness, show only limited associations with functional impairment and disease progression at the individual level [10]. Recently, it has become increasingly evident that morphometric properties of a given brain region are closely linked to the structural features of other functionally connected regions. This suggests that inter-individual differences in the structure of specific brain areas often covary with those of other regions, a pattern known as structural covariance. In this context, SCN analysis has emerged as a useful approach for examining coordinated patterns of brain morphology [10]. Previous SCN studies have shown that patients with PD exhibit deviations from the typical small-world topology seen in healthy individuals, including reduced global efficiency and altered modular structure [11], [12]. These alterations are already detectable in PD with normal cognition (PD-CN) and become more pronounced in those with PD-MCI, particularly within frontoparietal and limbic systems involved in executive and memory functions [13]. To further investigate this continuum of structural network disruption, we performed SCN analysis based on cortical thickness in healthy controls (HC), patients with PD-CN, and those with PD-MCI. This study aimed to identify stepwise alterations in brain network organization across cognitive stages in PD and to elucidate structural network features associated with cognitive impairment.

## 2 Methods

### 2.1 Study population

This retrospective observational study included 16 HC, 20 patients with PD-CN, and 22 patients with PD-MCI, recruited from the Department of Neurology, Nagoya City University Hospital (Nagoya, Japan) between April 2016 and March 2018. All patients with PD met the Movement Disorder Society (MDS) Clinical Diagnostic Criteria for PD [14]. The inclusion criteria were a Clinical Dementia Rating score < 1, age 50–85 years, Hoehn and Yahr stage <5, availability of a 3 T T1-weighted MRI, and completion of a comprehensive neuropsychological battery including Montreal Cognitive Assessment (MoCA) and Mini-Mental State Examination (MMSE) within 3 months of MRI scanning. Exclusion criteria included head trauma, cerebrovascular or metabolic disorders, severe psychiatric illness, and MRI evidence of major structural abnormalities unrelated to PD, such as large territorial infarctions, intracranial tumors, or hydrocephalus; diagnosis of dementia according to MDS criteria for Parkinson's disease dementia (MDS-PDD), white-matter hyperintensities with a Fazekas scale score ≥ 2 on MRI review, prior neurosurgical intervention, or any contraindication to MRI, as well as cases with significant preprocessing errors. Healthy controls were age- and sex-matched community volunteers with no history of neurological or psychiatric disorders.

### 2.2 Behavioral and cognitive assessments

Motor severity was assessed using the MDS-Unified Parkinson's Disease Rating Scale (MDS-UPDRS) [14] after withholding antiparkinsonian medication for at least 12 h. Cognitive assessments included the Montreal Cognitive Assessment (MoCA), Mini-Mental State Examination (MMSE), Trail Making Test A/B, Digit Span Backward, and the Stroop Color-Word Test.

### 2.3 Diagnosis of PD-MCI and PD-CN

PD-MCI was diagnosed according to the level I criteria of the Movement Disorder Society Task Force (MDS-TF) [15]. The diagnosis required (1) objective cognitive deficits, defined as a MoCA score of ≤25 (with the standard education correction applied) or neuropsychological test results falling at least two standard deviations below age-, sex-, and education-adjusted normative means in two or more cognitive domains, and (2) subjective cognitive decline reported by the patient or an informant, indicated by a score of 1 or more on item 1 (cognitive impairment) of the MDS-UPDRS Part I. Individuals with a MoCA score of ≥26 and no subjective cognitive decline were classified as PD-CN.

### 2.4 Statistical analysis of clinical characteristics

Group differences in continuous variables across the three groups were assessed using the Kruskal–Wallis test, with multiple comparisons corrected using the Benjamini–Hochberg false discovery rate (FDR) procedure (q < 0.05); Bonferroni-adjusted *p*-values are provided in Table 1. Where significant group effects were identified, pairwise post-hoc comparisons were performed using the Mann–Whitney *U* test with FDR and Bonferroni correction. Categorical variables (e.g., sex) were compared using the chi-squared test. Comparisons of PD-specific clinical characteristics between the PD-CN and PD-MCI groups — including disease duration, Hoehn and Yahr stage, levodopa equivalent daily dose (LEDD), and motor severity as measured by the MDS-UPDRS Part III OFF score — were performed separately using the Mann–Whitney *U* test with FDR and Bonferroni correction.

**Table 1:** Clinical characteristics.

Variable	HC (*n* = 16)	PD-CN (*n* = 20)	PD-MCI (*n* = 22)	p-value (FDR q)	p-value (Bonferroni)
Age, years	70.50 (4.65)	70.75 (5.84)	72.27 (5.93)	0.420	1.000
Male/Female	9/7	12/8	11/11	0.805†	0.805†
Education, years	12.88 (2.39)	12.15 (1.98)	11.64 (2.52)	0.265	1.000
Disease duration, years	N/A	7.45 (3.50)	6.64 (4.19)	0.613	1.000
Hoehn and Yahr stage	N/A	2.35 (0.59)	2.45 (0.51)	0.613	1.000
LEDD, mg	N/A	347.85 (151.57)	375.95 (134.79)	0.613	1.000
MDS-UPDRS III OFF	N/A	26.00 (14.86)	28.59 (14.05)	0.613	1.000
MoCA	28.62 (1.02)	27.65 (2.43)	21.50 (1.90)	< 0.001	< 0.001
MMSE	29.12 (0.72)	28.40 (1.76)	24.09 (1.93)	< 0.001	< 0.001
TMT-A, sec	50.32 (19.08)	65.90 (24.92)	80.58 (32.64)	0.0065	0.028
TMT-B, sec	97.04 (23.20)	158.05 (52.44)	225.12 (92.56)	< 0.001	< 0.001
Digit Span backward	5.19 (2.07)	4.65 (2.03)	3.23 (1.02)	0.0065	0.039
Stroop Color-Word test	52.81 (21.78)	64.90 (24.25)	91.00 (42.03)	0.0065	0.035

Data are mean (standard deviation). P-values for continuous variables were computed using the Kruskal–Wallis test (three-group comparisons: HC, PD-CN, PD-MCI) or Mann–Whitney U test (PD-CN vs. PD-MCI only). *P*-values for continuous variables were adjusted using the Benjamini–Hochberg false discovery rate (FDR) method and Bonferroni correction (×8 for three-group comparisons; ×4 for PD-only comparisons). †Sex was analyzed using Pearson's chi-squared test without multiple comparison correction. HC, Healthy control; PD-CN, Parkinson's disease with normal cognition; PD-MCI, Parkinson's disease with mild cognitive impairment; LEDD, levodopa equivalent daily dose; MDS-UPDRS III OFF, Movement DisorderSociety-Unified Parkinson's Disease Rating Scale part III off-state score; MoCA, Montreal cognitive assessment; MMSE, Mini-mental state examination; TMT, Trail making test.

### 2.5 Image acquisition

All participants underwent brain MRI using a 3-Tesla scanner (Magnetom Skyra, Siemens Healthcare, Erlangen, Germany) with a 32-channel head coil. 3D T1-weighted structural images were obtained using a magnetization-prepared rapid gradient echo sequence. Imaging parameters were as follows: repetition time = 7.3 ms, echo time = 2.4 ms, flip angle = 9°, inversion time = 900 ms, shot interval = 1900 ms, field of view = 256 × 256 × 176 mm^3^, matrix size = 256 × 256, and slice thickness = 1 mm, resulting in isotropic voxel resolution of 1 mm^3^.

### 2.6 Data preprocessing

Image preprocessing was conducted using the Computational Anatomy Toolbox (CAT12) implemented in SPM12. This included bias correction, tissue segmentation, surface reconstruction, and cortical thickness estimation. Cortical thickness values were extracted for 72 regions based on the Desikan-Killiany atlas (ROI_aparc_DK40). Regions that yielded missing values (NaN) or were labeled as “unknown” in any participant were excluded. As a result, 68 cortical regions were retained for subsequent network analysis. To account for demographic variability, cortical thickness values were normalized using the residuals from linear regression models controlling for age, sex, years of education, and intracranial volume (ICV).

### 2.7 Group-wise cortical thickness correlation

To evaluate group-level differences in the overall distribution of structural correlation values, we calculated the pairwise Pearson correlation coefficients between all cortical regions for each group. The resulting group-wise structural covariance matrices were used to visualize the distribution of correlation values.

### 2.8 Structural covariance network construction and graph analysis

SCNs were constructed separately for each group from Pearson correlation matrices of cortical thickness residuals after regressing out age, sex, education, and ICV. Across sparsity thresholds of 0.15–0.40 in steps of 0.01 [12], binarized undirected networks were generated by retaining the strongest absolute correlations as edges. Global metrics were computed within the largest connected component (LCC), including average clustering coefficient (ACC), average path length (APL), global efficiency (GE), local efficiency (LE), modularity (Q), and mean betweenness (BC), closeness (CC), and eigenvector (EC) centrality [6], [10], [16], [17]; small-world sigma and edge density were computed descriptively. Q used as a global graph metric was estimated using greedy modularity optimization implemented in NetworkX, while Louvain community detection was applied separately to visualize group-wise modular organization. Each metric was summarized as the area under the metric-vs-sparsity curve (AUC), normalized by the range width (0.25). Between-group differences in AUC were assessed using a bootstrap resampling framework (*N* = 1000) [12]: subjects within each group were resampled with replacement, group-level SCNs were reconstructed, and AUCs were compared by two-sample Welch's *t*-tests across 24 pairwise comparisons (3 group pairs × 8 inferential metrics; sigma and edge density were not tested), with Bonferroni and FDR-BH (q < 0.05) correction. This framework respects the group-level nature of SCN construction and avoids pseudo-replication across thresholds. For nodal centrality (BC, CC, EC), all 68 ROIs were retained across bootstrap iterations and isolated nodes were assigned zero centrality rather than excluded by LCC extraction. To visualize regional differences, a direction-coded SignedScore (−log10(q) × sign(Δ)) was computed for each ROI and projected onto the fsaverage cortical surface (FreeSurfer; https://surfer.nmr.mgh.harvard.edu/). Graph-theoretical metrics were computed using the NetworkX Python library [18].

### 2.9 Sensitivity analysis

Two sensitivity analyses were performed: (1) the bootstrap-AUC framework was repeated within a restricted sparsity range of 0.25–0.40 (16 thresholds), at which LCC sizes converge across groups; and (2) cortical thickness residuals for PD patients were recomputed including disease duration and LEDD as additional covariates, while the HC residual model was unchanged.

### 2.10 Cortical thickness–cognition correlation analysis

To explore the clinical relevance of structural network alterations at the individual level, Spearman rank correlations were computed between regional residual cortical thickness (68 Desikan–Killiany atlas ROIs) and cognitive scores (MoCA and MMSE), separately within three cohorts: the pooled PD group (PD-CN and PD-MCI; *n* = 42), PD-CN alone (*n* = 20), and PD-MCI alone (*n* = 22). Multiple comparisons within each cohort–cognitive score combination were corrected using the Benjamini–Hochberg FDR procedure (q < 0.05).

### 2.11 Modular organization

To investigate the modular architecture of the structural covariance networks, community detection was performed using the Louvain algorithm across sparsity thresholds ranging from 0.15 to 0.40 [6]. To visualize the progression of modular organization across groups, alluvial plots at a sparsity threshold of 0.30 were generated based on the results of Louvain community detection [6]. Node-wise community structure was assessed using the Louvain algorithm at a sparsity threshold of 0.30. As community labels derived from modularity optimization are arbitrary, direct label correspondence across groups was not assumed.

## 3 Results

### 3.1 Participant characteristics

A total of 58 participants were included: 16 HC, 20 patients with PD-CN, and 22 patients with PD-MCI. Clinical characteristics are summarized in Table 1. Age, sex distribution, and years of education did not differ across groups. Disease duration, Hoehn and Yahr stage, LEDD, and MDS-UPDRS Part III OFF score did not differ between PD-CN and PD-MCI (all q ≥ 0.613; all Bonferroni *p* = 1.000). MoCA, MMSE, TMT-A/B, the Stroop color-word test, and digit span backward differed significantly across groups after both FDR and Bonferroni correction.

### 3.2 Distribution of inter-regional correlation coefficients

**Fig. 1:**
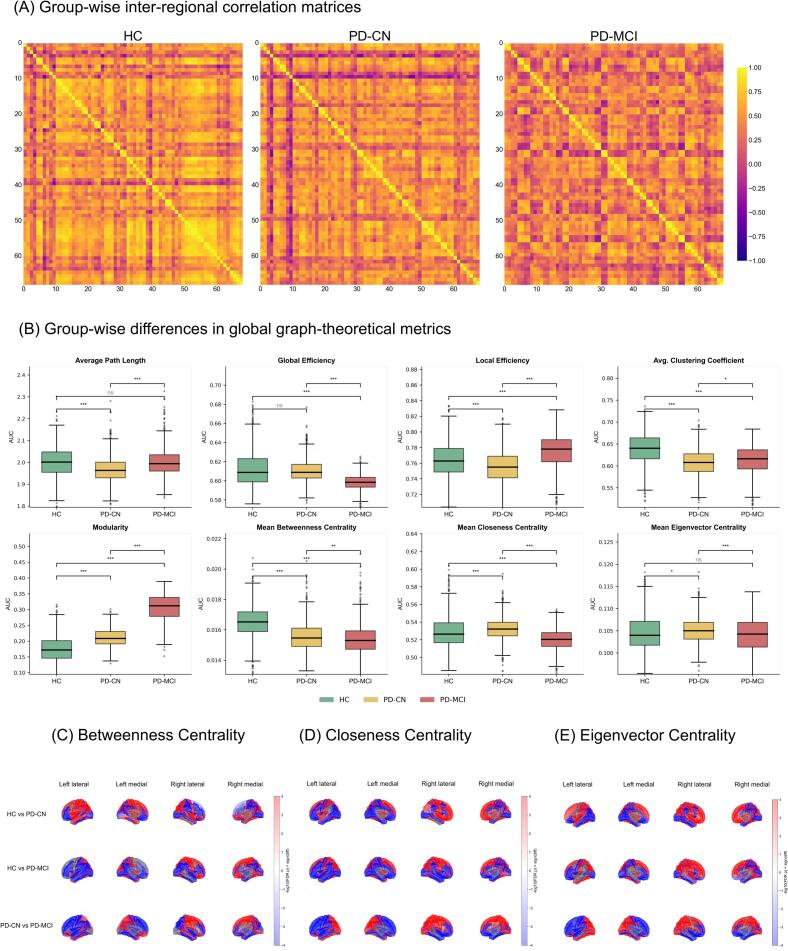
Structural covariance network organization across cognitive stages. (A) Group-wise inter-regional correlation matrices of cortical thickness for healthy controls (HC), Parkinson's disease with normal cognition (PD-CN), and Parkinson's disease with mild cognitive impairment (PD-MCI). Each axis represents cortical regions indexed numerically. The correspondence between ROI numbers and anatomical region names is provided in Supplementary Table 1. (B) Group-wise differences in global graph-theoretical metrics derived from structural covariance networks. Global metrics include average clustering coefficient, average path length, global efficiency, local efficiency, modularity, mean betweenness centrality, mean closeness centrality, and mean eigenvector centrality. Bootstrap-resampled AUC distributions are shown for each group, with *N* = 1000 bootstrap iterations per group. AUC values were computed across sparsity thresholds ranging from 0.15 to 0.40. Boxes represent the interquartile range with central lines indicating medians, whiskers extend to 1.5× the interquartile range, and gray points show outliers. Box colors indicate group membership (HC: green; PD-CN: yellow; PD-MCI: red). Pairwise group comparisons were performed using two-sample Welch's *t*-tests on bootstrap AUC distributions with Bonferroni correction (24 comparisons; *** *p* < 0.001, ** *p* < 0.01, * *p* < 0.05; ns: not significant). (C–E) Surface-based visualization of nodal centrality alterations across pairwise group comparisons (HC vs PD-CN, HC vs PD-MCI, PD-CN vs PD-MCI). Lateral and medial views of the left (L) and right (R) hemispheres are displayed. (C) Betweenness centrality, (D) closeness centrality, and (E) eigenvector centrality. Color scales represent the signed negative logarithm of the false discovery rate (FDR)-corrected *p*-values (−log₁₀[FDR-adjusted p] × sign of group difference), where warm colors indicate higher centrality in the *sec*ond group named in each pairwise comparison, whereas cool colors indicate lower centrality in that group. (For interpretation of the references to color in this figure legend, the reader is referred to the web version of this article.)

All groups showed a single-peaked distribution centered on positive correlation values, and the PD-MCI group exhibited a pronounced shift toward lower correlation values compared with the HC and PD-CN groups. The group-wise inter-regional correlation matrices are shown in Fig. 1A. To improve readability, brain regions are indexed numerically on both axes. The corresponding ROI names for each number are listed in Supplementary Table 1.

### 3.3 Global network metrics

Using the bootstrap-AUC framework (*N* = 1000 iterations per group), Q increased progressively across HC, PD-CN, and PD-MCI (all Bonferroni-corrected *p* < 0.001). GE was significantly reduced in PD-MCI compared with both HC and PD-CN (both *p* < 0.001), whereas the HC vs PD-CN difference did not survive Bonferroni correction (*p* = 0.110). APL showed a non-monotonic pattern, with shorter paths in PD-CN than in both HC and PD-MCI, whereas PD-MCI did not differ from HC after correction. ACC was lower in both PD groups than in HC (both *p* < 0.001), with a small but significant PD-CN vs PD-MCI difference (*p* = 0.042). LE also showed a non-monotonic pattern, with PD-CN lower than HC (p < 0.001) and PD-MCI higher than both HC and PD-CN (both p < 0.001). Mean BC, CC, and EC also showed significant group differences in several pairwise comparisons. These results are summarized in Fig. 1B and Table 2. The restricted-sparsity sensitivity analysis (sparsity 0.25–0.40; Supplementary Table 2) yielded largely consistent results, with three exceptions involving ACC, APL, and Mean Eigenvector Centrality. The PD-specific covariate sensitivity analysis preserved the relative PD-CN vs PD-MCI ordering for five of six evaluated metrics; only ACC showed a nominal direction reversal corresponding to a negligible primary-analysis difference (ΔAUC <0.001). LCC node counts at each sparsity threshold are reported in Supplementary Table 3; LCC size was not fully uniform at the lowest sparsity threshold (HC and PD-CN: 54 nodes; PD-MCI: 67 nodes), but all groups retained ≥61 nodes (≥ 89.7%) at sparsity ≥0.25.

**Table 2:** Bootstrap-Resampled AUC Comparisons of Global Network Metrics.

Variable	HC AUC, mean (SD)	PD-CN AUC, mean (SD)	PD-MCI AUC, mean (SD)	HC vs PD-CN p-value (Bonferroni)	HC vs PD-MCI p-value (Bonferroni)	PD-CN vs PD-MCI p-value (Bonferroni)
Average Clustering Coefficient (ACC)	0.6382 (0.0354)	0.6070 (0.0306)	0.6115 (0.0339)	<0.001	<0.001	0.042
Average Path Length (APL)	2.0001 (0.0738)	1.9670 (0.0581)	1.9983 (0.0622)	<0.001	1.000	<0.001
Global Efficiency (GE)	0.6126 (0.0183)	0.6106 (0.0117)	0.5983 (0.0080)	0.110	<0.001	<0.001
Local Efficiency (LE)	0.7641 (0.0221)	0.7548 (0.0208)	0.7750 (0.0220)	<0.001	<0.001	<0.001
Modularity (Q)	0.1762 (0.0399)	0.2113 (0.0292)	0.3058 (0.0423)	<0.001	<0.001	<0.001
Mean Betweenness Centrality (BC)	0.0165 (0.0010)	0.0155 (0.0009)	0.0154 (0.0010)	<0.001	<0.001	0.001
Mean Closeness Centrality (CC)	0.5289 (0.0180)	0.5322 (0.0127)	0.5200 (0.0124)	<0.001	<0.001	<0.001
Mean Eigenvector Centrality (EC)	0.1046 (0.0039)	0.1051 (0.0029)	0.1041 (0.0036)	0.049	0.099	<0.001

Data are area under the sparsity-vs-metric curve (AUC), normalized by range width (0.25), expressed as mean (standard deviation) from N = 1000 bootstrap iterations (sparsity range 0.15–0.40, 26 thresholds). Pairwise p-values were corrected using the Bonferroni method (24 comparisons: 3 group pairs × 8 metrics; α/24). Group comparisons were performed using two-sample Welch's t-tests. HC, healthy controls; PD-CN, Parkinson's disease with normal cognition; PD-MCI, Parkinson's disease with mild cognitive impairment; Q, modularity; GE, global efficiency; APL, average path length; ACC, average clustering coefficient; LE, local efficiency; BC, betweenness centrality; CC, closeness centrality; EC, eigenvector centrality.

### 3.4 Nodal centrality

Nodal centrality analyses revealed widespread group differences across cortical regions. After FDR correction (q < 0.05) within each pairwise comparison, significant differences were observed for BC in 34 left-hemispheric and 33 right-hemispheric regions, and for both CC and EC in 34 regions in each hemisphere. Representative regions included temporal, parietal, orbitofrontal, cingulate, and insular cortices. Cortical surface projections of these alterations are shown in Fig. 1C–E, and the complete list of significant regions is provided in Supplementary Table 4.

### 3.5 Modular organization and community detection

**Fig. 2:**
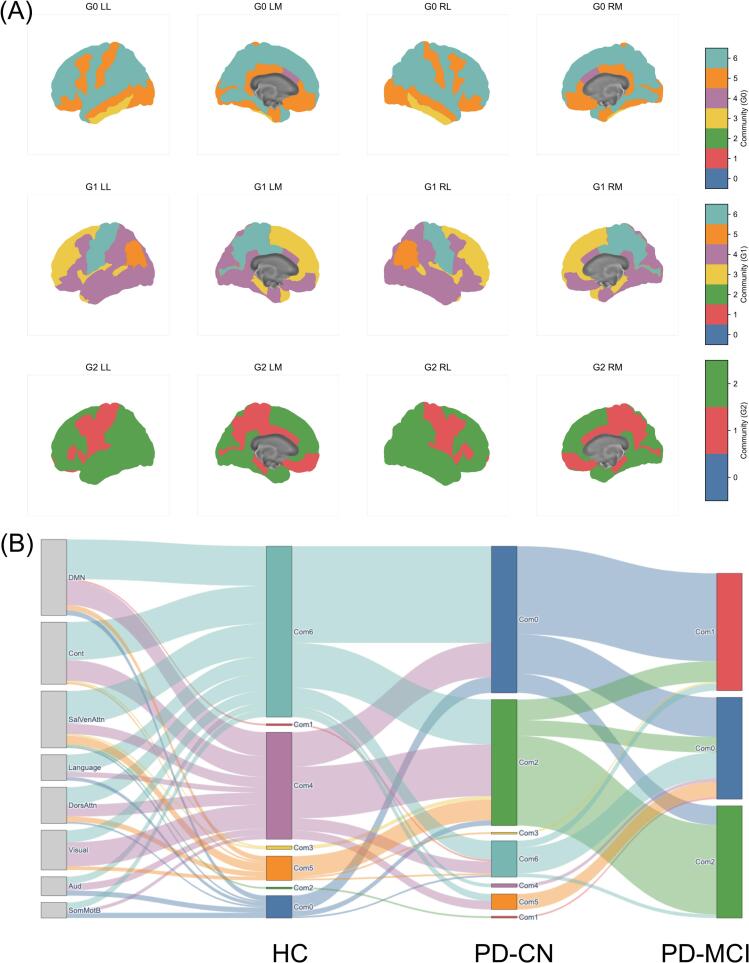
Alterations in modular organization and community structure across groups. (A) Group-wise modular organization of structural covariance networks projected onto the cortical surface. G0, G1, and G2 correspond to healthy controls (HC), Parkinson's disease with normal cognition (PD-CN), and Parkinson's disease with mild cognitive impairment (PD-MCI), respectively. LL and RL indicate left lateral and right lateral views, whereas LM and RM indicate left medial and right medial views. Community assignments were derived using the Louvain algorithm at a sparsity threshold of 0.30. Colors represent distinct communities identified within each group. The number and spatial distribution of modules differ across G0, G1, and G2. (B) Alluvial plots based on a sparsity threshold of 0.30 revealed that key regions, including the superior frontal gyrus and precuneus, changed community affiliation in PD-MCI. Cortical regions are categorized according to canonical functional networks, including the default mode network (DMN), frontoparietal control network (Cont), salience/ventral attention network (SalVenAttn), language network (Language), dorsal attention network (DorsAttn), somatomotor network B (SomMotB), auditory network (Aud), and visual network (Visual).

The structural covariance network exhibited a well-defined modular organization in the HC group, characterized by multiple spatially segregated communities distributed across frontal, parietal, and occipital cortices. In the PD-CN group, the overall modular architecture was largely preserved, although inter-module boundaries appeared less distinct. In contrast, the PD-MCI group showed a marked alteration in modular organization, with nodes converging into a smaller number of spatially extended modules encompassing widespread cortical regions. Surface projections of community structures are shown in Fig. 2A. Alluvial plots visualized transitions in community assignments across groups, highlighting pronounced reorganization in the PD-MCI group. Notably, several cortical regions typically associated with canonical functional systems, including the default mode, control, and dorsal attention networks, tended to be reassigned into fewer dominant modules. These patterns of modular reorganization, involving regions such as the superior frontal gyrus and precuneus, are illustrated in Fig. 2B.

### 3.6 Cortical thickness–cognition correlations

Spearman rank correlations between regional residual cortical thickness and cognitive scores identified four significant associations (FDR q < 0.05). In the pooled PD cohort (*n* = 42), greater cortical thickness in the bilateral superior temporal gyrus (STG) was significantly associated with higher MoCA scores (left STG: ρ = 0.502, q = 0.024; right STG: ρ = 0.527, q = 0.023). In the PD-MCI subgroup (*n* = 22), significant negative correlations were observed between cortical thickness and cognitive scores in the right lateral orbitofrontal cortex with MoCA (ρ = −0.685, q = 0.029) and in the left temporal pole with MMSE (ρ = −0.696, q = 0.022). No significant correlations were observed in PD-CN alone.

## 4 Discussion

We examined alterations in SCNs across HC, patients with PD-CN, and those with PD-MCI using graph-theoretical and community detection approaches. Based on cortical thickness–derived covariance networks, we evaluated both global and nodal-level properties, focusing on modular organization to characterize large-scale community structure and centrality measures to assess the relative importance of individual brain regions. Our findings suggest stage-dependent reorganization of brain network topology across cognitive stages in PD, characterized by reduced global efficiency, disrupted modular organization, and widespread changes in nodal centrality. These results indicate that SCN analysis is sensitive to network-level alterations associated with cognitive impairment in PD and provides insights into large-scale network reorganization accompanying cognitive decline.

At the global network level, Q showed a progressive, monotonic increase across the cognitive spectrum, indicating increasingly fragmented community structure as cognitive status declines. GE was reduced in PD-MCI compared with both HC and PD-CN, whereas the HC vs PD-CN difference did not survive Bonferroni correction, suggesting that long-range integrative capacity is most clearly impaired at the MCI stage. APL showed a non-monotonic pattern—PD-CN exhibited shorter paths than HC, while PD-MCI reverted to HC-comparable levels—consistent with stage-specific compensatory reorganization in PD-CN followed by subsequent network reorganization and reduced integration in PD-MCI. ACC was reduced in both PD groups compared with HC. Previous graph-theoretical studies in PD have reported alterations in both segregation and integration properties of brain networks. Segregation metrics, including ACC, LE, Q, and σ, reflect the extent to which networks are organized into locally clustered communities. Compared with healthy controls, patients with PD show reductions in ACC and LE in both structural and functional networks, whereas Q and σ may remain relatively preserved at early disease stages [19]. In contrast, patients with PD-related cognitive impairment often show more widespread and severe network abnormalities compared with PD-CN [20], [21], although regional increases in segregation metrics have occasionally been reported in PD-MCI, possibly reflecting transient compensatory reorganization [20]. In the present study, the LCC-based bootstrap analysis revealed a non-monotonic LE pattern: PD-CN exhibited lower LE than HC (0.755 ± 0.021 vs. 0.764 ± 0.022; *p* < 0.001), whereas PD-MCI showed significantly higher LE than both HC and PD-CN (0.775 ± 0.022; both Bonferroni-corrected p < 0.001). This U-shaped profile across the cognitive spectrum may reflect a compensatory increase in local clustering in PD-MCI in the context of impaired long-range integration, consistent with prior reports of regional segregation increases in PD-related cognitive impairment [20]. Integration metrics, such as characteristic APL and GE, index the efficiency of information transfer across the network. In prior studies, increased APL and reduced GE have frequently been reported in PD-CN and PD-MCI compared with HC [21], [22], [23]. However, prior studies have reported heterogeneous findings, with some showing the greatest integration deficits in PD-CN [22], while others report no global differences [24] or even reduced normalized characteristic path length in PD-CN [20]. In light of these prior findings, our observation of a monotonic increase in Q and selective reduction in GE at the MCI stage, together with the non-monotonic APL pattern, supports the notion of stage-dependent reorganization of large-scale brain networks associated with cognitive impairment in PD.

Beyond changes in global network analysis, we observed marked alterations in modular organization across cognitive stages in PD. Using the Louvain community detection algorithm and alluvial plots to characterize network community structure, HC exhibited a well-defined modular architecture with spatially segregated communities. In PD-CN, the overall modular structure was largely preserved, although boundaries between modules appeared less distinct. In contrast, PD-MCI showed a convergence of nodes into a smaller number of spatially extended modules encompassing widespread cortical regions, indicating a reorganization of large-scale community structure. Such alterations in modular organization are consistent with previous SCN studies employing similar approaches [6]. For example, compared with healthy controls, patients with dementia with Lewy bodies show severe disruption of community organization, with previously coherent modules becoming redistributed across networks [6]. These findings support the notion that loss of modular segregation reflects impaired organization of large-scale structural networks and may underlie cognitive deficits in Lewy body–related disorders. In this context, the modular reorganization observed in PD-MCI in the present study may reflect both reduced network specialization and compensatory integration across cortical systems accompanying cognitive decline.

Nodal centrality measures characterize different aspects of regional importance: BC reflects hub-mediated communication, CC reflects nodal proximity within the network, and EC reflects influence based on connections to highly connected regions. In this study, broadly comparable spatial extents of nodal alterations were observed across BC, CC, and EC, suggesting multifaceted reconfiguration of network topology, particularly at the PD-MCI stage. Previous studies across the PD cognitive continuum describe stage- and region-specific centrality changes, with focal compensatory increases in BC at early stages and reductions within key associative and sensorimotor hubs as cognitive impairment progresses [20], [22], [25], [26]. The widespread BC changes in PD-MCI observed here likely reflect increased vulnerability of integrative hubs. Reports of EC alterations have been less consistent in prior studies, and the broad EC changes identified here suggest a novel aspect of hierarchical network disruption associated with cognitive impairment in PD. The widespread CC changes also indicate that altered global nodal proximity is already evident at the PD-MCI stage, accompanying rather than simply following the emergence of hub-related reconfiguration—paralleling prior findings in dementia with Lewy bodies, where reductions in CC have been attributed to diffuse disruption of global distance structure [6]. Together, these findings support a model of multifaceted network topology disruption in PD-MCI.

Complementing the network-level findings, individual-level Spearman correlation analyses revealed that greater cortical thickness in the bilateral STG was significantly associated with higher MoCA scores in the pooled PD cohort. This finding is consistent with the well-established role of the STG in language and social cognition, and aligns with prior reports of STG thinning as a correlate of cognitive decline in PD [27]. However, the STG was not among the regions showing the most prominent nodal centrality changes at the network level, suggesting that regional cortical thickness and network centrality may capture partially distinct aspects of disease-related structural change. In the PD-MCI subgroup, negative correlations between cortical thickness and cognitive performance were identified in the right lateral orbitofrontal cortex and left temporal pole. Although statistically significant after FDR correction, the counterintuitive direction—greater thickness associated with lower cognitive scores—may reflect compensatory cortical reorganization. Given the modest PD-MCI sample size (*n* = 22), these negative associations should be considered exploratory and require replication in larger cohorts.

Several limitations should be acknowledged. First, the cross-sectional design precludes inference regarding the temporal evolution of network alterations across disease stages. Second, SCNs derived from cortical thickness reflect inter-individual statistical associations rather than direct anatomical or functional connectivity, and should be interpreted as indirect markers of large-scale network organization. Third, network metrics may be influenced by parcellation, network density, and thresholding strategy; LCC sizes were not fully uniform at the lowest sparsity thresholds (0.15–0.24), although the restricted-sparsity sensitivity analysis (0.25–0.40) supported the robustness of the main findings. Fourth, modest sample sizes—particularly for HC (*n* = 16) and PD-CN (*n* = 20)—may limit precision of estimated correlations and reduce statistical power, and multicenter replication in larger cohorts will be essential to confirm generalizability. Future longitudinal studies and integration with diffusion- and functional-MRI–based networks will be valuable for clarifying the mechanisms underlying network reorganization and cognitive decline in PD.

In conclusion, this study demonstrates that cognitive impairment in PD is associated with stage-dependent alterations in SCN organization, characterized not only by reduced network integration but also by disrupted hub-related centrality and altered modular architecture. The widespread alterations in BC, CC, and EC at the PD-MCI stage support a model of multifaceted network disruption involving hub-mediated information flow, global nodal proximity, and hierarchical network organization. These findings highlight the utility of network-based approaches for elucidating large-scale brain reorganization associated with cognitive decline in PD.

## CRediT authorship contribution statement

**Haruto Shibata:** Writing – original draft, Visualization, Validation, Software, Project administration, Methodology, Investigation, Formal analysis. **Yuto Uchida:** Writing – review & editing, Project administration, Funding acquisition, Data curation, Conceptualization. **Keita Sakurai:** Writing – review & editing, Visualization, Validation, Supervision, Software, Resources, Project administration, Methodology, Investigation, Formal analysis, Conceptualization. **Ken Tanaka:** Writing – review & editing, Supervision, Software, Methodology, Formal analysis, Conceptualization. **Yuya Kano:** Writing – review & editing, Investigation. **Noriyuki Matsukawa:** Writing – review & editing, Project administration.

## Ethics approval and consent to participate

This study was conducted in accordance with the Declaration of Helsinki. The study protocol was approved by the Institutional Review Board of Nagoya City University Hospital. Written informed consent was obtained from all participants.

## Funding

This work was supported by 10.13039/501100001691JSPS KAKENHI, Grant-in-Aid for Scientific Research (C) (Grant Number 22 K07520 to Yuto Uchida).

## Declaration of competing interest

The authors declare that they have no known competing financial interests or personal relationships that could have appeared to influence the work reported in this paper.

## Data Availability

The datasets generated and analyzed during the current study are not publicly available due to ethical and privacy restrictions associated with patient data but are available from the corresponding author upon reasonable request, subject to institutional approval.

## References

[1] Chaudhuri K.R., Healy D.G., Schapira A.H. (2006). Non-motor symptoms of Parkinson’s disease: diagnosis and management. Lancet Neurol..

[2] Rong S., Li Y., Li B. (2021). Meynert nucleus-related cortical thinning in Parkinson’s disease with mild cognitive impairment. Quant. Imaging Med. Surg..

[3] Jellinger K.A. (2023). Pathobiology of cognitive impairment in Parkinson disease: challenges and outlooks. Int. J. Mol. Sci..

[4] Mak E., Bergsland N., Dwyer M.G., Zivadinov R., Kandiah N. (2014). Subcortical atrophy is associated with cognitive impairment in mild Parkinson disease: a combined investigation of volumetric changes, cortical thickness, and vertex-based shape analysis. AJNR Am. J. Neuroradiol..

[5] Huang X., He Q., Ruan X. (2024). Structural connectivity from DTI to predict mild cognitive impairment in de novo Parkinson’s disease. Neuroimage Clin..

[6] Nicastro N., Mak E., Surendranathan A., Rittman T., Rowe J.B., O’Brien J.T. (2021). Altered structural connectivity networks in dementia with lewy bodies. Brain Imag. Behav..

[7] Amboni M., Tessitore A., Esposito F. (2015). Resting-state functional connectivity associated with mild cognitive impairment in Parkinson’s disease. J. Neurol..

[8] Piramide N., De Micco R., Siciliano M., Silvestro M., Tessitore A. (2024). Resting-state functional MRI approaches to Parkinsonisms and related dementia. Curr. Neurol. Neurosci. Rep..

[9] Chen X., Liu M., Wu Z., Cheng H. (2020). Topological abnormalities of functional brain network in early-stage Parkinson’s disease patients with mild cognitive impairment. Front. Neurosci..

[10] Mongay-Ochoa N., Gonzalez-Escamilla G., Fleischer V. (2025). Structural covariance analysis for neurodegenerative and neuroinflammatory brain disorders. Brain.

[11] Chen Y.S., Chen H.L., Lu C.H. (2021). The corticolimbic structural covariance network as an early predictive biosignature for cognitive impairment in Parkinson’s disease. Sci. Rep..

[12] Xu J., Zhang J., Zhang J. (2017). Abnormalities in structural covariance of cortical gyrification in Parkinson’s disease. Front. Neuroanat..

[13] Liu R.P., Lin G.L., Ma L.L. (2024). Changes of brain structure and structural covariance networks in Parkinson’s disease associated cognitive impairment. Front. Aging Neurosci..

[14] Postuma R.B., Berg D., Stern M. (2015). MDS clinical diagnostic criteria for Parkinson’s disease. Mov. Disord..

[15] Litvan I., Goldman J.G., Tröster A.I. (2012). Diagnostic criteria for mild cognitive impairment in Parkinson’s disease: movement disorder society task force guidelines. Mov. Disord..

[16] Hu Y., Xu J., Xiong J., Lv K., Geng D. (2025). Alterations of gray matter volume and structural covariance network in unilateral frontal lobe low-grade gliomas. BMC Med. Imaging.

[17] Wang Y., Chen S., Zhang P., Zhai Z., Chen Z., Li Z. (2024). Cortical structural network characteristics in non-cognitive impairment end-stage renal disease. Front. Neurosci..

[18] Hagberg A.A., Schult D.A., Swart P.J., Varoquaux G., Vaught T., Millman J. (2008). Proceedings of the 7th Python in Science Conference (SciPy 2008). Pasadena (CA).

[19] Vancea R., Simonyan K., Petracca M. (2019). Cognitive performance in mid-stage Parkinson’s disease: functional connectivity under chronic antiparkinson treatment. Brain Imag. Behav..

[20] Klobušiaková P., Mareček R., Fousek J., Výtvarová E., Rektorová I. (2019). Connectivity between brain networks dynamically reflects cognitive status of Parkinson’s disease: a longitudinal study. J. Alzheimer’s Dis.

[21] Suo X., Lei D., Li N. (2021). Topologically convergent and divergent morphological gray matter networks in early-stage Parkinson’s disease with and without mild cognitive impairment. Hum. Brain Mapp..

[22] Chen A., Li Y., Wang Z. (2022). Disrupted brain structural network connection in de novo Parkinson’s disease with rapid eye movement sleep behavior disorder. Front. Hum. Neurosci..

[23] Suo X., Lei D., Li N. (2022). Brain functional network abnormalities in parkinson’s disease with mild cognitive impairment. Cereb. Cortex.

[24] Inguanzo A., Segura B., Sala-Llonch R. (2021). Impaired structural connectivity in Parkinson’s disease patients with mild cognitive impairment: a study based on probabilistic tractography. Brain Connect..

[25] Chen P., Tang G., Wang Y. (2024). Spontaneous brain activity in the hippocampal regions could characterize cognitive impairment in patients with Parkinson’s disease. CNS Neurosci. Ther..

[26] Díez-Cirarda M., Strafella A.P., Kim J. (2017). Dynamic functional connectivity in Parkinson’s disease patients with mild cognitive impairment and normal cognition. Neuroimage Clin..

[27] Li L., Ji B., Zhao T., Cui X., Chen J., Wang Z. (2022). The structural changes of gray matter in Parkinson disease patients with mild cognitive impairments. PLoS One.

