# Supplementary material for: Structural covariance network alterations across the spectrum of cognitive status in Parkinson's disease
Source: Clin Park Relat Disord. 2026 May 26;14:100456. doi: 10.1016/j.prdoa.2026.100456 (PMC13251637; doi:10.1016/j.prdoa.2026.100456)
Supplement: Supplementary file 1 — Supplementary material 1 [file mmc1.docx]

| Supplementary Table 1. Region of interest number to name | |
| --- | --- |
| Number | Name |
| 0 | Banks of the Superior Temporal Sulcus (Left) |
| 1 | Banks of the Superior Temporal Sulcus (Right) |
| 2 | Caudal Anterior Cingulate (Left) |
| 3 | Caudal Anterior Cingulate (Right) |
| 4 | Caudal Middle Frontal (Left) |
| 5 | Caudal Middle Frontal (Right) |
| 6 | Cuneus (Left) |
| 7 | Cuneus (Right) |
| 8 | Entorhinal Cortex (Left) |
| 9 | Entorhinal Cortex (Right) |
| 10 | Fusiform (Left) |
| 11 | Fusiform (Right) |
| 12 | Inferior Parietal (Left) |
| 13 | Inferior Parietal (Right) |
| 14 | Inferior Temporal (Left) |
| 15 | Inferior Temporal (Right) |
| 16 | Isthmus Cingulate (Left) |
| 17 | Isthmus Cingulate (Right) |
| 18 | Lateral Occipital (Left) |
| 19 | Lateral Occipital (Right) |
| 20 | Lateral Orbitofrontal (Left) |
| 21 | Lateral Orbitofrontal (Right) |
| 22 | Lingual (Left) |
| 23 | Lingual (Right) |
| 24 | Medial Orbitofrontal (Left) |
| 25 | Medial Orbitofrontal (Right) |
| 26 | Middle Temporal (Left) |
| 27 | Middle Temporal (Right) |
| 28 | Parahippocampal (Left) |
| 29 | Parahippocampal (Right) |
| 30 | Paracentral (Left) |
| 31 | Paracentral (Right) |
| 32 | Pars Opercularis (Left) |
| 33 | Pars Opercularis (Right) |
| 34 | Pars Orbitalis (Left) |
| 35 | Pars Orbitalis (Right) |
| 36 | Pars Triangularis (Left) |
| 37 | Pars Triangularis (Right) |
| 38 | Pericalcarine (Left) |
| 39 | Pericalcarine (Right) |
| 40 | Postcentral (Left) |
| 41 | Postcentral (Right) |
| 42 | Posterior Cingulate (Left) |
| 43 | Posterior Cingulate (Right) |
| 44 | Precentral (Left) |
| 45 | Precentral (Right) |
| 46 | Precuneus (Left) |
| 47 | Precuneus (Right) |
| 48 | Rostral Anterior Cingulate (Left) |
| 49 | Rostral Anterior Cingulate (Right) |
| 50 | Rostral Middle Frontal (Left) |
| 51 | Rostral Middle Frontal (Right) |
| 52 | Superior Frontal (Left) |
| 53 | Superior Frontal (Right) |
| 54 | Superior Parietal (Left) |
| 55 | Superior Parietal (Right) |
| 56 | Superior Temporal (Left) |
| 57 | Superior Temporal (Right) |
| 58 | Supramarginal (Left) |
| 59 | Supramarginal (Right) |
| 60 | Frontal Pole (Left) |
| 61 | Frontal Pole (Right) |
| 62 | Temporal Pole (Left) |
| 63 | Temporal Pole (Right) |
| 64 | Transverse Temporal (Left) |
| 65 | Transverse Temporal (Right) |
| 66 | Insula (Left) |
| 67 | Insula (Right) |
