# Supplementary material for: Structural covariance network alterations across the spectrum of cognitive status in Parkinson's disease
Source: Clin Park Relat Disord. 2026 May 26;14:100456. doi: 10.1016/j.prdoa.2026.100456 (PMC13251637; doi:10.1016/j.prdoa.2026.100456)
Supplement: Supplementary file 2 — Supplementary material 2 [file mmc2.docx]

| Supplementary Table 2. Sensitivity Analysis: Bootstrap-Resampled AUC Comparisons of Global Network Metrics (Sparsity 0.25–0.40) | | | | | | | | | | |
| --- | --- | --- | --- | --- | --- | --- | --- | --- | --- | --- |
| Variable | HC  AUC, mean (SD) | PD-CN  AUC, mean (SD) | | | PD-MCI  AUC, mean (SD) | HC vs PD-CN  p-value (Bonferroni) | HC vs PD-MCI  p-value (Bonferroni) | PD-CN vs PD-MCI  p-value (Bonferroni) | | |
| Average Clustering Coefficient (ACC) | 0.6641 (0.0363) | 0.6351 (0.0313) | | | 0.6379 (0.0373) | <0.001 | <0.001 | 1.000 | | |
| Average Path Length (APL) | 1.8817 (0.0710) | 1.8418 (0.0573) | | | 1.8240 (0.0539) | <0.001 | <0.001 | <0.001 | | |
| Global Efficiency (GE) | 0.6425 (0.0155) | 0.6427 (0.0105) | | | 0.6396 (0.0079) | 1.000 | <0.001 | <0.001 | | |
| Local Efficiency (LE) | 0.7890 (0.0220) | 0.7802 (0.0213) | | | 0.8018 (0.0218) | <0.001 | <0.001 | <0.001 | | |
| Modularity (Q) | 0.1554 (0.0357) | 0.1851 (0.0277) | | | 0.2794 (0.0411) | <0.001 | <0.001 | <0.001 | | |
| Mean Betweenness Centrality (BC) | 0.0140 (0.0011) | 0.0131 (0.0009) | | | 0.0125 (0.0008) | <0.001 | <0.001 | <0.001 | | |
| Mean Closeness Centrality (CC) | 0.5579 (0.0174) | 0.5636 (0.0132) | | | 0.5614 (0.0126) | <0.001 | <0.001 | 0.005 | | |
| Mean Eigenvector Centrality (EC) | 0.1053 (0.0031) | 0.1060 (0.0024) | | | 0.1075 (0.0032) | <0.001 | <0.001 | <0.001 | | |
| Data are area under the sparsity-vs-metric curve (AUC), normalised by range width (0.15), expressed as mean (standard deviation) from N = 1,000 bootstrap iterations (sensitivity analysis; sparsity range 0.25–0.40, 16 thresholds). Pairwise p-values were corrected using the Bonferroni method (24 comparisons: 3 group pairs × 8 metrics; α/24). Group comparisons were performed using two-sample Welch’s t-tests. HC, healthy controls; PD-CN, Parkinson’s disease with normal cognition; PD-MCI, Parkinson’s disease with mild cognitive impairment; Q, modularity; GE, global efficiency; APL, average path length; ACC, average clustering coefficient; LE, local efficiency; BC, betweenness centrality; CC, closeness centrality; EC, eigenvector centrality. | | | | | | | | | | |
|  |  | |  |  | | | | |  |  |
