# Supplementary material for: Structural covariance network alterations across the spectrum of cognitive status in Parkinson's disease
Source: Clin Park Relat Disord. 2026 May 26;14:100456. doi: 10.1016/j.prdoa.2026.100456 (PMC13251637; doi:10.1016/j.prdoa.2026.100456)
Supplement: Supplementary file 3 — Supplementary material 3 [file mmc3.docx]

| Supplementary Table 3. Largest connected component (LCC) node counts by sparsity threshold | | | |
| --- | --- | --- | --- |
| Sparsity  threshold | HC  (LCC nodes) | PD-CN  (LCC nodes) | PD-MCI  (LCC nodes) |
| 0.15 | 54 | 54 | 67 |
| 0.16 | 57 | 54 | 67 |
| 0.17 | 59 | 59 | 67 |
| 0.18 | 61 | 59 | 67 |
| 0.19 | 61 | 60 | 67 |
| 0.20 | 62 | 60 | 67 |
| 0.21 | 62 | 60 | 67 |
| 0.22 | 62 | 61 | 67 |
| 0.23 | 62 | 61 | 67 |
| 0.24 | 62 | 61 | 67 |
| 0.25 | 62 | 61 | 68 |
| 0.26 | 63 | 61 | 68 |
| 0.27 | 63 | 62 | 68 |
| 0.28 | 63 | 62 | 68 |
| 0.29 | 65 | 64 | 68 |
| 0.30 | 65 | 65 | 68 |
| 0.31 | 65 | 65 | 68 |
| 0.32 | 65 | 66 | 68 |
| 0.33 | 66 | 66 | 68 |
| 0.34 | 67 | 66 | 68 |
| 0.35 | 67 | 67 | 68 |
| 0.36 | 67 | 67 | 68 |
| 0.37 | 67 | 67 | 68 |
| 0.38 | 67 | 67 | 68 |
| 0.39 | 67 | 67 | 68 |
| 0.40 | 67 | 67 | 68 |

Number of nodes included in the largest connected component (LCC) of the structural covariance network at each sparsity threshold (0.15–0.40), for each diagnostic group. The maximum possible LCC size is 68 nodes (all cortical ROIs). HC, healthy controls; PD-CN, Parkinson’s disease with normal cognition; PD-MCI, Parkinson’s disease with mild cognitive impairment; LCC, largest connected component; ROI, region of interest.
