# Supplementary material for: Structural covariance network alterations across the spectrum of cognitive status in Parkinson's disease
Source: Clin Park Relat Disord. 2026 May 26;14:100456. doi: 10.1016/j.prdoa.2026.100456 (PMC13251637; doi:10.1016/j.prdoa.2026.100456)
Supplement: Supplementary file 4 — Supplementary material 4 [file mmc4.docx]

| Supplementary Table 4. Complete list of cortical regions showing significant differences in nodal centrality between groups (FDR q < 0.05) | | | | | | |
| --- | --- | --- | --- | --- | --- | --- |
| **Metric** | **Hemisphere** | **Comparison** | **ROI Name** | **Mean Diff** | **FDR q-value** | **Signed Score** |
| BC | left | HC vs PD-CN | Superior parietal cortex | 0.0289 | < 1e-300 | > 300 |
| BC | left | HC vs PD-CN | Fusiform gyrus | 0.0183 | 5.48e-276 | 275.26 |
| BC | left | HC vs PD-CN | Pars opercularis | 0.0122 | 6.06e-225 | 224.22 |
| BC | left | HC vs PD-CN | Insula | 0.0128 | 2.05e-206 | 205.69 |
| BC | left | HC vs PD-CN | Transverse temporal gyrus | -0.0139 | 3.04e-147 | -146.52 |
| BC | left | HC vs PD-CN | Banks of the superior temporal sulcus | -0.0153 | 1.28e-139 | -138.89 |
| BC | left | HC vs PD-CN | Superior frontal gyrus | 0.0088 | 1.04e-125 | 124.98 |
| BC | left | HC vs PD-CN | Inferior temporal gyrus | -0.0134 | 5.80e-122 | -121.24 |
| BC | left | HC vs PD-CN | Frontal pole | 0.0102 | 2.08e-104 | 103.68 |
| BC | left | HC vs PD-CN | Middle temporal gyrus | 0.0101 | 1.73e-101 | 100.76 |
| BC | left | HC vs PD-CN | Medial orbitofrontal cortex | -0.0109 | 2.85e-92 | -91.54 |
| BC | left | HC vs PD-CN | Lateral orbitofrontal cortex | -0.0103 | 1.09e-83 | -82.96 |
| BC | left | HC vs PD-CN | Posterior cingulate cortex | -0.0097 | 1.35e-79 | -78.87 |
| BC | left | HC vs PD-CN | Lateral occipital cortex | -0.0081 | 6.49e-76 | -75.19 |
| BC | left | HC vs PD-CN | Postcentral gyrus | 0.0072 | 6.53e-71 | 70.18 |
| BC | left | HC vs PD-CN | Pars orbitalis | -0.0054 | 1.10e-64 | -63.96 |
| BC | left | HC vs PD-CN | Rostral middle frontal gyrus | -0.0049 | 8.46e-57 | -56.07 |
| BC | left | HC vs PD-CN | Isthmus of the cingulate gyrus | -0.0055 | 1.77e-54 | -53.75 |
| BC | left | HC vs PD-CN | Precuneus | -0.0052 | 4.28e-46 | -45.37 |
| BC | left | HC vs PD-CN | Paracentral lobule | 0.0060 | 8.11e-40 | 39.09 |
| BC | left | HC vs PD-CN | Inferior parietal cortex | 0.0042 | 2.60e-26 | 25.59 |
| BC | left | HC vs PD-CN | Pars triangularis | -0.0035 | 2.44e-20 | -19.61 |
| BC | left | HC vs PD-CN | Precentral gyrus | 0.0032 | 2.45e-19 | 18.61 |
| BC | left | HC vs PD-CN | Supramarginal gyrus | 0.0039 | 2.22e-15 | 14.65 |
| BC | left | HC vs PD-CN | Rostral anterior cingulate cortex | -0.0023 | 2.62e-11 | -10.58 |
| BC | left | HC vs PD-CN | Superior temporal gyrus | 0.0025 | 8.97e-11 | 10.05 |
| BC | left | HC vs PD-CN | Entorhinal cortex | 0.0016 | 1.24e-10 | 9.91 |
| BC | left | HC vs PD-CN | Caudal anterior cingulate cortex | 0.0017 | 2.06e-8 | 7.69 |
| BC | left | HC vs PD-CN | Cuneus | -0.0024 | 6.65e-7 | -6.18 |
| BC | left | HC vs PD-CN | Parahippocampal gyrus | -0.0009 | 0.001 | -2.90 |
| BC | left | HC vs PD-CN | Lingual gyrus | 0.0009 | 0.041 | 1.39 |
| BC | left | HC vs PD-MCI | Superior parietal cortex | 0.0301 | < 1e-300 | > 300 |
| BC | left | HC vs PD-MCI | Superior temporal gyrus | -0.0185 | 9.56e-272 | -271.02 |
| BC | left | HC vs PD-MCI | Rostral middle frontal gyrus | -0.0239 | 1.89e-264 | -263.72 |
| BC | left | HC vs PD-MCI | Pars opercularis | -0.0214 | 1.51e-226 | -225.82 |
| BC | left | HC vs PD-MCI | Fusiform gyrus | 0.0142 | 2.89e-187 | 186.54 |
| BC | left | HC vs PD-MCI | Pars orbitalis | -0.0102 | 3.59e-181 | -180.44 |
| BC | left | HC vs PD-MCI | Paracentral lobule | 0.0110 | 1.08e-127 | 126.97 |
| BC | left | HC vs PD-MCI | Parahippocampal gyrus | -0.0108 | 6.53e-126 | -125.19 |
| BC | left | HC vs PD-MCI | Supramarginal gyrus | -0.0148 | 2.39e-121 | -120.62 |
| BC | left | HC vs PD-MCI | Transverse temporal gyrus | -0.0089 | 2.06e-119 | -118.69 |
| BC | left | HC vs PD-MCI | Temporal pole | 0.0121 | 2.00e-110 | 109.70 |
| BC | left | HC vs PD-MCI | Frontal pole | 0.0097 | 4.76e-98 | 97.32 |
| BC | left | HC vs PD-MCI | Rostral anterior cingulate cortex | -0.0060 | 1.10e-80 | -79.96 |
| BC | left | HC vs PD-MCI | Insula | 0.0074 | 1.60e-76 | 75.80 |
| BC | left | HC vs PD-MCI | Lateral occipital cortex | -0.0091 | 3.30e-63 | -62.48 |
| BC | left | HC vs PD-MCI | Caudal middle frontal gyrus | -0.0082 | 5.79e-63 | -62.24 |
| BC | left | HC vs PD-MCI | Lingual gyrus | -0.0070 | 1.75e-61 | -60.76 |
| BC | left | HC vs PD-MCI | Postcentral gyrus | -0.0090 | 5.68e-54 | -53.25 |
| BC | left | HC vs PD-MCI | Pericalcarine cortex | 0.0042 | 1.59e-44 | 43.80 |
| BC | left | HC vs PD-MCI | Banks of the superior temporal sulcus | -0.0053 | 3.77e-28 | -27.42 |
| BC | left | HC vs PD-MCI | Inferior temporal gyrus | -0.0056 | 3.80e-28 | -27.42 |
| BC | left | HC vs PD-MCI | Isthmus of the cingulate gyrus | -0.0040 | 1.56e-27 | -26.81 |
| BC | left | HC vs PD-MCI | Medial orbitofrontal cortex | -0.0042 | 1.84e-26 | -25.73 |
| BC | left | HC vs PD-MCI | Pars triangularis | -0.0039 | 6.39e-23 | -22.19 |
| BC | left | HC vs PD-MCI | Cuneus | 0.0039 | 2.83e-19 | 18.55 |
| BC | left | HC vs PD-MCI | Inferior parietal cortex | -0.0054 | 6.79e-16 | -15.17 |
| BC | left | HC vs PD-MCI | Entorhinal cortex | -0.0021 | 3.65e-13 | -12.44 |
| BC | left | HC vs PD-MCI | Precuneus | -0.0022 | 1.05e-9 | -8.98 |
| BC | left | HC vs PD-MCI | Posterior cingulate cortex | 0.0013 | 1.35e-4 | 3.87 |
| BC | left | HC vs PD-MCI | Middle temporal gyrus | -0.0020 | 2.70e-4 | -3.57 |
| BC | left | HC vs PD-MCI | Caudal anterior cingulate cortex | -0.0008 | 0.014 | -1.87 |
| BC | left | PD-CN vs PD-MCI | Pars opercularis | -0.0336 | < 1e-300 | < -300 |
| BC | left | PD-CN vs PD-MCI | Superior temporal gyrus | -0.0209 | < 1e-300 | < -300 |
| BC | left | PD-CN vs PD-MCI | Rostral middle frontal gyrus | -0.0190 | 2.27e-192 | -191.64 |
| BC | left | PD-CN vs PD-MCI | Supramarginal gyrus | -0.0187 | 1.37e-171 | -170.86 |
| BC | left | PD-CN vs PD-MCI | Postcentral gyrus | -0.0162 | 7.27e-151 | -150.14 |
| BC | left | PD-CN vs PD-MCI | Temporal pole | 0.0133 | 1.03e-143 | 142.99 |
| BC | left | PD-CN vs PD-MCI | Posterior cingulate cortex | 0.0110 | 1.69e-112 | 111.77 |
| BC | left | PD-CN vs PD-MCI | Parahippocampal gyrus | -0.0099 | 7.38e-105 | -104.13 |
| BC | left | PD-CN vs PD-MCI | Lateral orbitofrontal cortex | 0.0105 | 1.66e-103 | 102.78 |
| BC | left | PD-CN vs PD-MCI | Middle temporal gyrus | -0.0122 | 1.43e-102 | -101.85 |
| BC | left | PD-CN vs PD-MCI | Superior frontal gyrus | -0.0091 | 1.52e-78 | -77.82 |
| BC | left | PD-CN vs PD-MCI | Banks of the superior temporal sulcus | 0.0100 | 2.51e-71 | 70.60 |
| BC | left | PD-CN vs PD-MCI | Lingual gyrus | -0.0079 | 3.10e-68 | -67.51 |
| BC | left | PD-CN vs PD-MCI | Cuneus | 0.0062 | 9.49e-56 | 55.02 |
| BC | left | PD-CN vs PD-MCI | Caudal middle frontal gyrus | -0.0076 | 2.70e-55 | -54.57 |
| BC | left | PD-CN vs PD-MCI | Insula | -0.0054 | 3.36e-53 | -52.47 |
| BC | left | PD-CN vs PD-MCI | Pars orbitalis | -0.0049 | 2.82e-52 | -51.55 |
| BC | left | PD-CN vs PD-MCI | Entorhinal cortex | -0.0037 | 2.99e-50 | -49.52 |
| BC | left | PD-CN vs PD-MCI | Inferior parietal cortex | -0.0096 | 1.40e-45 | -44.85 |
| BC | left | PD-CN vs PD-MCI | Medial orbitofrontal cortex | 0.0067 | 3.46e-45 | 44.46 |
| BC | left | PD-CN vs PD-MCI | Paracentral lobule | 0.0050 | 5.98e-41 | 40.22 |
| BC | left | PD-CN vs PD-MCI | Inferior temporal gyrus | 0.0077 | 7.01e-38 | 37.15 |
| BC | left | PD-CN vs PD-MCI | Pericalcarine cortex | 0.0040 | 2.81e-35 | 34.55 |
| BC | left | PD-CN vs PD-MCI | Rostral anterior cingulate cortex | -0.0037 | 1.37e-25 | -24.86 |
| BC | left | PD-CN vs PD-MCI | Caudal anterior cingulate cortex | -0.0025 | 1.29e-24 | -23.89 |
| BC | left | PD-CN vs PD-MCI | Fusiform gyrus | -0.0041 | 1.48e-24 | -23.83 |
| BC | left | PD-CN vs PD-MCI | Precentral gyrus | -0.0038 | 2.55e-23 | -22.59 |
| BC | left | PD-CN vs PD-MCI | Transverse temporal gyrus | 0.0050 | 3.55e-22 | 21.45 |
| BC | left | PD-CN vs PD-MCI | Precuneus | 0.0030 | 1.09e-18 | 17.96 |
| BC | left | PD-CN vs PD-MCI | Isthmus of the cingulate gyrus | 0.0016 | 1.17e-4 | 3.93 |
| BC | left | PD-CN vs PD-MCI | Superior parietal cortex | 0.0012 | 0.001 | 2.90 |
| BC | right | HC vs PD-CN | Banks of the superior temporal sulcus | -0.0223 | 9.06e-318 | -317.04 |
| BC | right | HC vs PD-CN | Caudal middle frontal gyrus | -0.0201 | 1.14e-218 | -217.94 |
| BC | right | HC vs PD-CN | Pars opercularis | -0.0148 | 5.63e-208 | -207.25 |
| BC | right | HC vs PD-CN | Pars orbitalis | 0.0164 | 7.32e-201 | 200.14 |
| BC | right | HC vs PD-CN | Isthmus of the cingulate gyrus | 0.0128 | 3.59e-186 | 185.45 |
| BC | right | HC vs PD-CN | Fusiform gyrus | 0.0146 | 6.61e-152 | 151.18 |
| BC | right | HC vs PD-CN | Supramarginal gyrus | -0.0144 | 1.26e-146 | -145.90 |
| BC | right | HC vs PD-CN | Precentral gyrus | 0.0166 | 6.18e-144 | 143.21 |
| BC | right | HC vs PD-CN | Precuneus | -0.0123 | 4.52e-139 | -138.34 |
| BC | right | HC vs PD-CN | Superior parietal cortex | 0.0069 | 4.83e-111 | 110.32 |
| BC | right | HC vs PD-CN | Pars triangularis | -0.0082 | 1.59e-104 | -103.80 |
| BC | right | HC vs PD-CN | Posterior cingulate cortex | -0.0123 | 1.16e-98 | -97.94 |
| BC | right | HC vs PD-CN | Parahippocampal gyrus | 0.0074 | 2.66e-85 | 84.58 |
| BC | right | HC vs PD-CN | Rostral anterior cingulate cortex | 0.0074 | 1.03e-70 | 69.99 |
| BC | right | HC vs PD-CN | Paracentral lobule | -0.0076 | 1.05e-70 | -69.98 |
| BC | right | HC vs PD-CN | Medial orbitofrontal cortex | 0.0078 | 1.94e-49 | 48.71 |
| BC | right | HC vs PD-CN | Transverse temporal gyrus | -0.0064 | 1.22e-46 | -45.91 |
| BC | right | HC vs PD-CN | Lateral occipital cortex | -0.0044 | 5.63e-40 | -39.25 |
| BC | right | HC vs PD-CN | Entorhinal cortex | 0.0043 | 4.37e-31 | 30.36 |
| BC | right | HC vs PD-CN | Inferior parietal cortex | 0.0036 | 1.50e-27 | 26.82 |
| BC | right | HC vs PD-CN | Insula | 0.0039 | 7.11e-26 | 25.15 |
| BC | right | HC vs PD-CN | Cuneus | 0.0046 | 1.62e-22 | 21.79 |
| BC | right | HC vs PD-CN | Superior temporal gyrus | 0.0036 | 7.61e-21 | 20.12 |
| BC | right | HC vs PD-CN | Frontal pole | 0.0026 | 1.01e-14 | 14.00 |
| BC | right | HC vs PD-CN | Temporal pole | -0.0028 | 4.78e-14 | -13.32 |
| BC | right | HC vs PD-CN | Lingual gyrus | -0.0043 | 1.79e-12 | -11.75 |
| BC | right | HC vs PD-CN | Pericalcarine cortex | -0.0025 | 4.19e-11 | -10.38 |
| BC | right | HC vs PD-CN | Postcentral gyrus | -0.0023 | 6.20e-8 | -7.21 |
| BC | right | HC vs PD-CN | Middle temporal gyrus | -0.0027 | 2.55e-6 | -5.59 |
| BC | right | HC vs PD-CN | Lateral orbitofrontal cortex | -0.0021 | 5.39e-6 | -5.27 |
| BC | right | HC vs PD-CN | Rostral middle frontal gyrus | -0.0012 | 9.07e-6 | -5.04 |
| BC | right | HC vs PD-CN | Superior frontal gyrus | -0.0008 | 0.040 | -1.39 |
| BC | right | HC vs PD-MCI | Rostral middle frontal gyrus | -0.0190 | 2.62e-255 | -254.58 |
| BC | right | HC vs PD-MCI | Pars opercularis | -0.0153 | 2.10e-235 | -234.68 |
| BC | right | HC vs PD-MCI | Pars orbitalis | 0.0169 | 1.08e-219 | 218.97 |
| BC | right | HC vs PD-MCI | Inferior temporal gyrus | -0.0199 | 3.23e-202 | -201.49 |
| BC | right | HC vs PD-MCI | Insula | -0.0142 | 1.75e-199 | -198.76 |
| BC | right | HC vs PD-MCI | Precentral gyrus | 0.0197 | 1.43e-188 | 187.85 |
| BC | right | HC vs PD-MCI | Lingual gyrus | 0.0164 | 2.25e-159 | 158.65 |
| BC | right | HC vs PD-MCI | Isthmus of the cingulate gyrus | 0.0111 | 1.06e-139 | 138.98 |
| BC | right | HC vs PD-MCI | Posterior cingulate cortex | -0.0112 | 6.42e-130 | -129.19 |
| BC | right | HC vs PD-MCI | Fusiform gyrus | 0.0128 | 4.60e-113 | 112.34 |
| BC | right | HC vs PD-MCI | Entorhinal cortex | -0.0087 | 1.62e-85 | -84.79 |
| BC | right | HC vs PD-MCI | Pericalcarine cortex | -0.0074 | 1.61e-84 | -83.79 |
| BC | right | HC vs PD-MCI | Superior temporal gyrus | -0.0072 | 2.22e-79 | -78.65 |
| BC | right | HC vs PD-MCI | Pars triangularis | -0.0073 | 1.48e-69 | -68.83 |
| BC | right | HC vs PD-MCI | Lateral orbitofrontal cortex | 0.0055 | 5.64e-63 | 62.25 |
| BC | right | HC vs PD-MCI | Transverse temporal gyrus | 0.0047 | 2.20e-61 | 60.66 |
| BC | right | HC vs PD-MCI | Superior frontal gyrus | 0.0073 | 3.09e-53 | 52.51 |
| BC | right | HC vs PD-MCI | Supramarginal gyrus | -0.0078 | 1.86e-48 | -47.73 |
| BC | right | HC vs PD-MCI | Lateral occipital cortex | -0.0043 | 3.01e-35 | -34.52 |
| BC | right | HC vs PD-MCI | Temporal pole | -0.0046 | 9.60e-35 | -34.02 |
| BC | right | HC vs PD-MCI | Superior parietal cortex | 0.0044 | 2.68e-30 | 29.57 |
| BC | right | HC vs PD-MCI | Paracentral lobule | 0.0033 | 4.89e-18 | 17.31 |
| BC | right | HC vs PD-MCI | Postcentral gyrus | 0.0022 | 1.84e-9 | 8.74 |
| BC | right | HC vs PD-MCI | Inferior parietal cortex | -0.0026 | 6.98e-9 | -8.16 |
| BC | right | HC vs PD-MCI | Precuneus | -0.0021 | 1.05e-7 | -6.98 |
| BC | right | HC vs PD-MCI | Rostral anterior cingulate cortex | 0.0023 | 2.99e-7 | 6.52 |
| BC | right | HC vs PD-MCI | Frontal pole | -0.0016 | 2.08e-6 | -5.68 |
| BC | right | HC vs PD-MCI | Medial orbitofrontal cortex | 0.0022 | 2.12e-5 | 4.67 |
| BC | right | PD-CN vs PD-MCI | Banks of the superior temporal sulcus | 0.0228 | < 1e-300 | > 300 |
| BC | right | PD-CN vs PD-MCI | Insula | -0.0181 | < 1e-300 | < -300 |
| BC | right | PD-CN vs PD-MCI | Lingual gyrus | 0.0206 | 2.72e-317 | 316.56 |
| BC | right | PD-CN vs PD-MCI | Entorhinal cortex | -0.0130 | 3.82e-234 | -233.42 |
| BC | right | PD-CN vs PD-MCI | Rostral middle frontal gyrus | -0.0178 | 1.68e-226 | -225.78 |
| BC | right | PD-CN vs PD-MCI | Caudal middle frontal gyrus | 0.0203 | 1.15e-209 | 208.94 |
| BC | right | PD-CN vs PD-MCI | Paracentral lobule | 0.0109 | 1.91e-192 | 191.72 |
| BC | right | PD-CN vs PD-MCI | Inferior temporal gyrus | -0.0195 | 1.99e-191 | -190.70 |
| BC | right | PD-CN vs PD-MCI | Superior temporal gyrus | -0.0108 | 1.20e-169 | -168.92 |
| BC | right | PD-CN vs PD-MCI | Transverse temporal gyrus | 0.0112 | 3.93e-129 | 128.41 |
| BC | right | PD-CN vs PD-MCI | Precuneus | 0.0102 | 2.08e-104 | 103.68 |
| BC | right | PD-CN vs PD-MCI | Parahippocampal gyrus | -0.0079 | 1.47e-88 | -87.83 |
| BC | right | PD-CN vs PD-MCI | Lateral orbitofrontal cortex | 0.0075 | 1.24e-75 | 74.91 |
| BC | right | PD-CN vs PD-MCI | Superior frontal gyrus | 0.0081 | 6.76e-54 | 53.17 |
| BC | right | PD-CN vs PD-MCI | Inferior parietal cortex | -0.0062 | 1.65e-45 | -44.78 |
| BC | right | PD-CN vs PD-MCI | Frontal pole | -0.0042 | 1.93e-45 | -44.71 |
| BC | right | PD-CN vs PD-MCI | Medial orbitofrontal cortex | -0.0055 | 1.27e-37 | -36.90 |
| BC | right | PD-CN vs PD-MCI | Pericalcarine cortex | -0.0048 | 1.61e-35 | -34.79 |
| BC | right | PD-CN vs PD-MCI | Rostral anterior cingulate cortex | -0.0050 | 7.26e-33 | -32.14 |
| BC | right | PD-CN vs PD-MCI | Cuneus | -0.0053 | 2.94e-31 | -30.53 |
| BC | right | PD-CN vs PD-MCI | Supramarginal gyrus | 0.0066 | 1.74e-28 | 27.76 |
| BC | right | PD-CN vs PD-MCI | Postcentral gyrus | 0.0045 | 8.44e-22 | 21.07 |
| BC | right | PD-CN vs PD-MCI | Precentral gyrus | 0.0031 | 1.76e-15 | 14.75 |
| BC | right | PD-CN vs PD-MCI | Isthmus of the cingulate gyrus | -0.0018 | 4.78e-14 | -13.32 |
| BC | right | PD-CN vs PD-MCI | Superior parietal cortex | -0.0025 | 4.44e-12 | -11.35 |
| BC | right | PD-CN vs PD-MCI | Middle temporal gyrus | 0.0035 | 5.42e-12 | 11.27 |
| BC | right | PD-CN vs PD-MCI | Temporal pole | -0.0018 | 5.07e-7 | -6.30 |
| BC | right | PD-CN vs PD-MCI | Fusiform gyrus | -0.0018 | 3.63e-4 | -3.44 |
| BC | right | PD-CN vs PD-MCI | Pars triangularis | 0.0009 | 0.025 | 1.60 |
| CC | left | HC vs PD-CN | Pars opercularis | 0.0803 | < 1e-300 | > 300 |
| CC | left | HC vs PD-CN | Superior parietal cortex | 0.1001 | < 1e-300 | > 300 |
| CC | left | HC vs PD-CN | Transverse temporal gyrus | -0.0867 | 5.32e-319 | -318.27 |
| CC | left | HC vs PD-CN | Banks of the superior temporal sulcus | -0.1782 | 4.83e-312 | -311.32 |
| CC | left | HC vs PD-CN | Insula | 0.0813 | 1.07e-308 | 307.97 |
| CC | left | HC vs PD-CN | Lateral orbitofrontal cortex | -0.1570 | 1.26e-303 | -302.90 |
| CC | left | HC vs PD-CN | Lateral occipital cortex | -0.0926 | 2.02e-235 | -234.69 |
| CC | left | HC vs PD-CN | Superior frontal gyrus | 0.0673 | 6.27e-215 | 214.20 |
| CC | left | HC vs PD-CN | Cuneus | -0.1891 | 3.42e-214 | -213.47 |
| CC | left | HC vs PD-CN | Precuneus | -0.2242 | 2.64e-200 | -199.58 |
| CC | left | HC vs PD-CN | Pars orbitalis | -0.1039 | 6.57e-185 | -184.18 |
| CC | left | HC vs PD-CN | Posterior cingulate cortex | -0.1165 | 3.02e-182 | -181.52 |
| CC | left | HC vs PD-CN | Postcentral gyrus | 0.0620 | 9.19e-179 | 178.04 |
| CC | left | HC vs PD-CN | Fusiform gyrus | 0.0765 | 1.77e-173 | 172.75 |
| CC | left | HC vs PD-CN | Paracentral lobule | -0.0876 | 9.01e-108 | -107.05 |
| CC | left | HC vs PD-CN | Medial orbitofrontal cortex | -0.1269 | 1.05e-107 | -106.98 |
| CC | left | HC vs PD-CN | Inferior temporal gyrus | -0.0408 | 6.61e-86 | -85.18 |
| CC | left | HC vs PD-CN | Isthmus of the cingulate gyrus | -0.0574 | 6.11e-79 | -78.21 |
| CC | left | HC vs PD-CN | Pars triangularis | -0.0331 | 1.42e-46 | -45.85 |
| CC | left | HC vs PD-CN | Rostral middle frontal gyrus | -0.0205 | 1.14e-33 | -32.94 |
| CC | left | HC vs PD-CN | Pericalcarine cortex | -0.0601 | 9.65e-26 | -25.02 |
| CC | left | HC vs PD-CN | Caudal anterior cingulate cortex | -0.0515 | 8.72e-13 | -12.06 |
| CC | left | HC vs PD-CN | Precentral gyrus | -0.0166 | 1.56e-11 | -10.81 |
| CC | left | HC vs PD-CN | Rostral anterior cingulate cortex | -0.0431 | 5.26e-10 | -9.28 |
| CC | left | HC vs PD-CN | Lingual gyrus | -0.0175 | 2.52e-9 | -8.60 |
| CC | left | HC vs PD-CN | Temporal pole | -0.0297 | 5.16e-9 | -8.29 |
| CC | left | HC vs PD-CN | Inferior parietal cortex | -0.0079 | 1.37e-8 | -7.86 |
| CC | left | HC vs PD-CN | Caudal middle frontal gyrus | -0.0116 | 3.79e-8 | -7.42 |
| CC | left | HC vs PD-CN | Parahippocampal gyrus | -0.0323 | 2.18e-7 | -6.66 |
| CC | left | HC vs PD-CN | Entorhinal cortex | -0.0320 | 1.16e-6 | -5.94 |
| CC | left | HC vs PD-CN | Middle temporal gyrus | -0.0052 | 2.73e-4 | -3.56 |
| CC | left | HC vs PD-CN | Superior temporal gyrus | 0.0065 | 0.001 | 2.97 |
| CC | left | HC vs PD-MCI | Parahippocampal gyrus | -0.2263 | < 1e-300 | < -300 |
| CC | left | HC vs PD-MCI | Superior parietal cortex | 0.1395 | < 1e-300 | > 300 |
| CC | left | HC vs PD-MCI | Fusiform gyrus | 0.0729 | 1.84e-310 | 309.74 |
| CC | left | HC vs PD-MCI | Rostral middle frontal gyrus | -0.0647 | 3.11e-255 | -254.51 |
| CC | left | HC vs PD-MCI | Medial orbitofrontal cortex | -0.2029 | 2.57e-250 | -249.59 |
| CC | left | HC vs PD-MCI | Pars orbitalis | -0.1267 | 1.86e-246 | -245.73 |
| CC | left | HC vs PD-MCI | Rostral anterior cingulate cortex | -0.2207 | 3.75e-245 | -244.43 |
| CC | left | HC vs PD-MCI | Caudal anterior cingulate cortex | -0.2346 | 3.15e-244 | -243.50 |
| CC | left | HC vs PD-MCI | Transverse temporal gyrus | -0.0653 | 3.78e-234 | -233.42 |
| CC | left | HC vs PD-MCI | Entorhinal cortex | -0.1996 | 6.87e-229 | -228.16 |
| CC | left | HC vs PD-MCI | Superior frontal gyrus | 0.0461 | 1.10e-221 | 220.96 |
| CC | left | HC vs PD-MCI | Banks of the superior temporal sulcus | -0.1275 | 2.89e-201 | -200.54 |
| CC | left | HC vs PD-MCI | Lateral orbitofrontal cortex | -0.1026 | 2.19e-172 | -171.66 |
| CC | left | HC vs PD-MCI | Pars triangularis | -0.0629 | 1.88e-166 | -165.72 |
| CC | left | HC vs PD-MCI | Cuneus | -0.1509 | 3.13e-152 | -151.50 |
| CC | left | HC vs PD-MCI | Lateral occipital cortex | -0.0710 | 8.76e-152 | -151.06 |
| CC | left | HC vs PD-MCI | Lingual gyrus | -0.0577 | 1.20e-146 | -145.92 |
| CC | left | HC vs PD-MCI | Insula | 0.0376 | 1.58e-142 | 141.80 |
| CC | left | HC vs PD-MCI | Precuneus | -0.1648 | 4.79e-125 | -124.32 |
| CC | left | HC vs PD-MCI | Pars opercularis | -0.0260 | 2.42e-93 | -92.62 |
| CC | left | HC vs PD-MCI | Supramarginal gyrus | -0.0318 | 1.78e-90 | -89.75 |
| CC | left | HC vs PD-MCI | Inferior parietal cortex | 0.0296 | 6.84e-81 | 80.17 |
| CC | left | HC vs PD-MCI | Superior temporal gyrus | -0.0307 | 1.12e-67 | -66.95 |
| CC | left | HC vs PD-MCI | Caudal middle frontal gyrus | -0.0287 | 6.65e-53 | -52.18 |
| CC | left | HC vs PD-MCI | Middle temporal gyrus | -0.0205 | 2.45e-43 | -42.61 |
| CC | left | HC vs PD-MCI | Paracentral lobule | -0.0490 | 9.87e-40 | -39.01 |
| CC | left | HC vs PD-MCI | Precentral gyrus | -0.0279 | 4.45e-30 | -29.35 |
| CC | left | HC vs PD-MCI | Isthmus of the cingulate gyrus | -0.0367 | 1.27e-27 | -26.90 |
| CC | left | HC vs PD-MCI | Posterior cingulate cortex | -0.0411 | 1.88e-27 | -26.73 |
| CC | left | HC vs PD-MCI | Pericalcarine cortex | -0.0541 | 1.02e-23 | -22.99 |
| CC | left | HC vs PD-MCI | Inferior temporal gyrus | -0.0179 | 1.30e-23 | -22.89 |
| CC | left | HC vs PD-MCI | Temporal pole | 0.0484 | 1.30e-18 | 17.89 |
| CC | left | HC vs PD-MCI | Frontal pole | -0.0264 | 8.75e-7 | -6.06 |
| CC | left | HC vs PD-MCI | Postcentral gyrus | 0.0070 | 5.62e-5 | 4.25 |
| CC | left | PD-CN vs PD-MCI | Pars opercularis | -0.1064 | < 1e-300 | < -300 |
| CC | left | PD-CN vs PD-MCI | Parahippocampal gyrus | -0.1940 | 2.89e-205 | -204.54 |
| CC | left | PD-CN vs PD-MCI | Lateral orbitofrontal cortex | 0.0543 | 2.39e-197 | 196.62 |
| CC | left | PD-CN vs PD-MCI | Caudal anterior cingulate cortex | -0.1831 | 3.25e-187 | -186.49 |
| CC | left | PD-CN vs PD-MCI | Rostral anterior cingulate cortex | -0.1776 | 7.45e-184 | -183.13 |
| CC | left | PD-CN vs PD-MCI | Entorhinal cortex | -0.1676 | 4.38e-180 | -179.36 |
| CC | left | PD-CN vs PD-MCI | Postcentral gyrus | -0.0549 | 2.29e-175 | -174.64 |
| CC | left | PD-CN vs PD-MCI | Rostral middle frontal gyrus | -0.0442 | 2.63e-156 | -155.58 |
| CC | left | PD-CN vs PD-MCI | Banks of the superior temporal sulcus | 0.0507 | 4.33e-131 | 130.36 |
| CC | left | PD-CN vs PD-MCI | Posterior cingulate cortex | 0.0754 | 6.92e-131 | 130.16 |
| CC | left | PD-CN vs PD-MCI | Insula | -0.0437 | 1.89e-111 | -110.72 |
| CC | left | PD-CN vs PD-MCI | Inferior parietal cortex | 0.0376 | 8.30e-108 | 107.08 |
| CC | left | PD-CN vs PD-MCI | Precuneus | 0.0594 | 4.62e-106 | 105.34 |
| CC | left | PD-CN vs PD-MCI | Medial orbitofrontal cortex | -0.0760 | 1.26e-101 | -100.90 |
| CC | left | PD-CN vs PD-MCI | Superior temporal gyrus | -0.0372 | 9.94e-95 | -94.00 |
| CC | left | PD-CN vs PD-MCI | Supramarginal gyrus | -0.0339 | 7.71e-90 | -89.11 |
| CC | left | PD-CN vs PD-MCI | Superior parietal cortex | 0.0394 | 2.42e-65 | 64.62 |
| CC | left | PD-CN vs PD-MCI | Pars triangularis | -0.0298 | 1.34e-57 | -56.87 |
| CC | left | PD-CN vs PD-MCI | Temporal pole | 0.0780 | 1.12e-53 | 52.95 |
| CC | left | PD-CN vs PD-MCI | Pars orbitalis | -0.0228 | 1.90e-48 | -47.72 |
| CC | left | PD-CN vs PD-MCI | Lingual gyrus | -0.0402 | 3.09e-42 | -41.51 |
| CC | left | PD-CN vs PD-MCI | Paracentral lobule | 0.0386 | 1.78e-41 | 40.75 |
| CC | left | PD-CN vs PD-MCI | Cuneus | 0.0382 | 1.89e-41 | 40.72 |
| CC | left | PD-CN vs PD-MCI | Transverse temporal gyrus | 0.0213 | 3.89e-36 | 35.41 |
| CC | left | PD-CN vs PD-MCI | Lateral occipital cortex | 0.0215 | 4.61e-30 | 29.34 |
| CC | left | PD-CN vs PD-MCI | Superior frontal gyrus | -0.0212 | 7.89e-27 | -26.10 |
| CC | left | PD-CN vs PD-MCI | Inferior temporal gyrus | 0.0228 | 2.69e-26 | 25.57 |
| CC | left | PD-CN vs PD-MCI | Middle temporal gyrus | -0.0153 | 7.97e-24 | -23.10 |
| CC | left | PD-CN vs PD-MCI | Caudal middle frontal gyrus | -0.0171 | 6.60e-19 | -18.18 |
| CC | left | PD-CN vs PD-MCI | Isthmus of the cingulate gyrus | 0.0207 | 9.34e-13 | 12.03 |
| CC | left | PD-CN vs PD-MCI | Frontal pole | -0.0267 | 5.02e-10 | -9.30 |
| CC | left | PD-CN vs PD-MCI | Precentral gyrus | -0.0113 | 1.23e-8 | -7.91 |
| CC | right | HC vs PD-CN | Banks of the superior temporal sulcus | -0.1604 | < 1e-300 | < -300 |
| CC | right | HC vs PD-CN | Lingual gyrus | -0.1344 | < 1e-300 | < -300 |
| CC | right | HC vs PD-CN | Paracentral lobule | -0.2030 | < 1e-300 | < -300 |
| CC | right | HC vs PD-CN | Pars opercularis | -0.1062 | < 1e-300 | < -300 |
| CC | right | HC vs PD-CN | Pars orbitalis | 0.1211 | < 1e-300 | > 300 |
| CC | right | HC vs PD-CN | Isthmus of the cingulate gyrus | 0.2004 | 1.64e-281 | 280.78 |
| CC | right | HC vs PD-CN | Lateral occipital cortex | -0.0915 | 8.57e-225 | -224.07 |
| CC | right | HC vs PD-CN | Fusiform gyrus | 0.0943 | 3.51e-218 | 217.46 |
| CC | right | HC vs PD-CN | Rostral anterior cingulate cortex | 0.1537 | 9.16e-215 | 214.04 |
| CC | right | HC vs PD-CN | Posterior cingulate cortex | -0.1136 | 4.26e-206 | -205.37 |
| CC | right | HC vs PD-CN | Caudal middle frontal gyrus | -0.0508 | 4.59e-194 | -193.34 |
| CC | right | HC vs PD-CN | Precuneus | -0.1361 | 5.32e-181 | -180.27 |
| CC | right | HC vs PD-CN | Superior parietal cortex | 0.0523 | 2.73e-178 | 177.56 |
| CC | right | HC vs PD-CN | Supramarginal gyrus | -0.0506 | 3.39e-168 | -167.47 |
| CC | right | HC vs PD-CN | Medial orbitofrontal cortex | 0.0861 | 8.53e-122 | 121.07 |
| CC | right | HC vs PD-CN | Temporal pole | -0.1618 | 4.34e-112 | -111.36 |
| CC | right | HC vs PD-CN | Insula | 0.0557 | 3.14e-87 | 86.50 |
| CC | right | HC vs PD-CN | Pericalcarine cortex | -0.1265 | 3.25e-70 | -69.49 |
| CC | right | HC vs PD-CN | Transverse temporal gyrus | 0.0441 | 1.80e-68 | 67.74 |
| CC | right | HC vs PD-CN | Middle temporal gyrus | -0.0291 | 5.21e-66 | -65.28 |
| CC | right | HC vs PD-CN | Precentral gyrus | 0.0374 | 8.34e-65 | 64.08 |
| CC | right | HC vs PD-CN | Rostral middle frontal gyrus | 0.0225 | 3.39e-61 | 60.47 |
| CC | right | HC vs PD-CN | Superior temporal gyrus | 0.0338 | 4.10e-53 | 52.39 |
| CC | right | HC vs PD-CN | Pars triangularis | -0.0187 | 7.39e-40 | -39.13 |
| CC | right | HC vs PD-CN | Frontal pole | 0.0904 | 1.60e-36 | 35.79 |
| CC | right | HC vs PD-CN | Caudal anterior cingulate cortex | -0.0899 | 1.18e-33 | -32.93 |
| CC | right | HC vs PD-CN | Superior frontal gyrus | 0.0131 | 1.22e-23 | 22.91 |
| CC | right | HC vs PD-CN | Parahippocampal gyrus | 0.0527 | 4.90e-18 | 17.31 |
| CC | right | HC vs PD-CN | Postcentral gyrus | 0.0142 | 5.04e-15 | 14.30 |
| CC | right | HC vs PD-CN | Entorhinal cortex | -0.0461 | 8.00e-13 | -12.10 |
| CC | right | HC vs PD-CN | Lateral orbitofrontal cortex | 0.0236 | 2.95e-11 | 10.53 |
| CC | right | HC vs PD-CN | Cuneus | 0.0196 | 1.61e-10 | 9.79 |
| CC | right | HC vs PD-CN | Inferior parietal cortex | 0.0030 | 0.013 | 1.88 |
| CC | right | HC vs PD-MCI | Inferior temporal gyrus | -0.1646 | < 1e-300 | < -300 |
| CC | right | HC vs PD-MCI | Pars opercularis | -0.1020 | < 1e-300 | < -300 |
| CC | right | HC vs PD-MCI | Entorhinal cortex | -0.2415 | 1.64e-311 | -310.78 |
| CC | right | HC vs PD-MCI | Superior frontal gyrus | 0.0680 | 1.67e-303 | 302.78 |
| CC | right | HC vs PD-MCI | Insula | -0.0849 | 1.13e-292 | -291.95 |
| CC | right | HC vs PD-MCI | Superior parietal cortex | 0.0787 | 1.71e-256 | 255.77 |
| CC | right | HC vs PD-MCI | Parahippocampal gyrus | -0.1440 | 1.48e-216 | -215.83 |
| CC | right | HC vs PD-MCI | Posterior cingulate cortex | -0.1083 | 1.82e-193 | -192.74 |
| CC | right | HC vs PD-MCI | Caudal anterior cingulate cortex | -0.2136 | 1.57e-189 | -188.81 |
| CC | right | HC vs PD-MCI | Postcentral gyrus | 0.0523 | 2.16e-183 | 182.66 |
| CC | right | HC vs PD-MCI | Temporal pole | -0.2053 | 1.66e-175 | -174.78 |
| CC | right | HC vs PD-MCI | Transverse temporal gyrus | 0.0609 | 6.28e-167 | 166.20 |
| CC | right | HC vs PD-MCI | Pars orbitalis | 0.0816 | 1.92e-158 | 157.72 |
| CC | right | HC vs PD-MCI | Paracentral lobule | -0.1090 | 3.49e-136 | -135.46 |
| CC | right | HC vs PD-MCI | Frontal pole | -0.1230 | 2.21e-118 | -117.66 |
| CC | right | HC vs PD-MCI | Caudal middle frontal gyrus | 0.0427 | 3.18e-117 | 116.50 |
| CC | right | HC vs PD-MCI | Pericalcarine cortex | -0.1642 | 5.99e-114 | -113.22 |
| CC | right | HC vs PD-MCI | Lateral occipital cortex | -0.0651 | 7.86e-111 | -110.10 |
| CC | right | HC vs PD-MCI | Precentral gyrus | 0.0539 | 1.85e-110 | 109.73 |
| CC | right | HC vs PD-MCI | Inferior parietal cortex | 0.0340 | 4.26e-99 | 98.37 |
| CC | right | HC vs PD-MCI | Fusiform gyrus | 0.0490 | 3.83e-95 | 94.42 |
| CC | right | HC vs PD-MCI | Isthmus of the cingulate gyrus | 0.1046 | 1.11e-78 | 77.95 |
| CC | right | HC vs PD-MCI | Middle temporal gyrus | -0.0280 | 8.36e-70 | -69.08 |
| CC | right | HC vs PD-MCI | Banks of the superior temporal sulcus | -0.0524 | 3.84e-63 | -62.42 |
| CC | right | HC vs PD-MCI | Rostral middle frontal gyrus | -0.0188 | 2.43e-51 | -50.62 |
| CC | right | HC vs PD-MCI | Lateral orbitofrontal cortex | -0.0319 | 4.61e-47 | -46.34 |
| CC | right | HC vs PD-MCI | Supramarginal gyrus | -0.0203 | 2.61e-36 | -35.58 |
| CC | right | HC vs PD-MCI | Superior temporal gyrus | -0.0161 | 2.29e-32 | -31.64 |
| CC | right | HC vs PD-MCI | Precuneus | -0.0456 | 3.37e-27 | -26.47 |
| CC | right | HC vs PD-MCI | Medial orbitofrontal cortex | -0.0218 | 9.92e-21 | -20.00 |
| CC | right | HC vs PD-MCI | Rostral anterior cingulate cortex | 0.0252 | 1.31e-13 | 12.88 |
| CC | right | HC vs PD-MCI | Pars triangularis | -0.0076 | 9.26e-8 | -7.03 |
| CC | right | HC vs PD-MCI | Cuneus | -0.0089 | 9.96e-5 | -4.00 |
| CC | right | HC vs PD-MCI | Lingual gyrus | 0.0093 | 0.003 | 2.59 |
| CC | right | PD-CN vs PD-MCI | Banks of the superior temporal sulcus | 0.1080 | < 1e-300 | > 300 |
| CC | right | PD-CN vs PD-MCI | Caudal middle frontal gyrus | 0.0935 | < 1e-300 | > 300 |
| CC | right | PD-CN vs PD-MCI | Lingual gyrus | 0.1437 | < 1e-300 | > 300 |
| CC | right | PD-CN vs PD-MCI | Insula | -0.1406 | < 1e-300 | < -300 |
| CC | right | PD-CN vs PD-MCI | Paracentral lobule | 0.0939 | 5.90e-267 | 266.23 |
| CC | right | PD-CN vs PD-MCI | Precuneus | 0.0905 | 2.69e-253 | 252.57 |
| CC | right | PD-CN vs PD-MCI | Entorhinal cortex | -0.1954 | 9.36e-225 | -224.03 |
| CC | right | PD-CN vs PD-MCI | Inferior temporal gyrus | -0.1641 | 1.16e-220 | -219.94 |
| CC | right | PD-CN vs PD-MCI | Parahippocampal gyrus | -0.1967 | 3.47e-209 | -208.46 |
| CC | right | PD-CN vs PD-MCI | Frontal pole | -0.2134 | 4.84e-209 | -208.32 |
| CC | right | PD-CN vs PD-MCI | Superior frontal gyrus | 0.0549 | 3.30e-186 | 185.48 |
| CC | right | PD-CN vs PD-MCI | Medial orbitofrontal cortex | -0.1079 | 2.25e-183 | -182.65 |
| CC | right | PD-CN vs PD-MCI | Rostral middle frontal gyrus | -0.0414 | 3.57e-142 | -141.45 |
| CC | right | PD-CN vs PD-MCI | Rostral anterior cingulate cortex | -0.1286 | 8.28e-136 | -135.08 |
| CC | right | PD-CN vs PD-MCI | Superior temporal gyrus | -0.0499 | 2.68e-102 | -101.57 |
| CC | right | PD-CN vs PD-MCI | Caudal anterior cingulate cortex | -0.1237 | 1.35e-92 | -91.87 |
| CC | right | PD-CN vs PD-MCI | Inferior parietal cortex | 0.0310 | 8.51e-87 | 86.07 |
| CC | right | PD-CN vs PD-MCI | Postcentral gyrus | 0.0381 | 2.36e-73 | 72.63 |
| CC | right | PD-CN vs PD-MCI | Fusiform gyrus | -0.0453 | 9.43e-70 | -69.03 |
| CC | right | PD-CN vs PD-MCI | Supramarginal gyrus | 0.0302 | 2.92e-66 | 65.53 |
| CC | right | PD-CN vs PD-MCI | Lateral orbitofrontal cortex | -0.0555 | 1.90e-57 | -56.72 |
| CC | right | PD-CN vs PD-MCI | Isthmus of the cingulate gyrus | -0.0958 | 7.34e-51 | -50.13 |
| CC | right | PD-CN vs PD-MCI | Superior parietal cortex | 0.0263 | 2.29e-33 | 32.64 |
| CC | right | PD-CN vs PD-MCI | Pars orbitalis | -0.0395 | 7.03e-33 | -32.15 |
| CC | right | PD-CN vs PD-MCI | Lateral occipital cortex | 0.0264 | 3.41e-28 | 27.47 |
| CC | right | PD-CN vs PD-MCI | Temporal pole | -0.0435 | 7.97e-24 | -23.10 |
| CC | right | PD-CN vs PD-MCI | Pericalcarine cortex | -0.0377 | 1.32e-18 | -17.88 |
| CC | right | PD-CN vs PD-MCI | Cuneus | -0.0285 | 5.92e-18 | -17.23 |
| CC | right | PD-CN vs PD-MCI | Pars triangularis | 0.0111 | 3.94e-14 | 13.41 |
| CC | right | PD-CN vs PD-MCI | Precentral gyrus | 0.0165 | 4.05e-13 | 12.39 |
| CC | right | PD-CN vs PD-MCI | Transverse temporal gyrus | 0.0169 | 1.60e-12 | 11.80 |
| CC | right | PD-CN vs PD-MCI | Pars opercularis | 0.0042 | 0.018 | 1.74 |
| CC | right | PD-CN vs PD-MCI | Posterior cingulate cortex | 0.0052 | 0.020 | 1.69 |
| EC | left | HC vs PD-CN | Banks of the superior temporal sulcus | -0.1177 | < 1e-300 | < -300 |
| EC | left | HC vs PD-CN | Lateral orbitofrontal cortex | -0.1017 | < 1e-300 | < -300 |
| EC | left | HC vs PD-CN | Superior parietal cortex | 0.0783 | < 1e-300 | > 300 |
| EC | left | HC vs PD-CN | Pars opercularis | 0.0720 | 7.60e-303 | 302.12 |
| EC | left | HC vs PD-CN | Insula | 0.0683 | 2.50e-269 | 268.60 |
| EC | left | HC vs PD-CN | Precuneus | -0.0855 | 1.58e-265 | -264.80 |
| EC | left | HC vs PD-CN | Postcentral gyrus | 0.0700 | 1.06e-263 | 262.97 |
| EC | left | HC vs PD-CN | Superior frontal gyrus | 0.0656 | 2.57e-257 | 256.59 |
| EC | left | HC vs PD-CN | Cuneus | -0.0664 | 1.60e-248 | -247.79 |
| EC | left | HC vs PD-CN | Lateral occipital cortex | -0.0691 | 6.51e-219 | -218.19 |
| EC | left | HC vs PD-CN | Pars orbitalis | -0.0598 | 5.74e-176 | -175.24 |
| EC | left | HC vs PD-CN | Transverse temporal gyrus | -0.0531 | 9.68e-176 | -175.01 |
| EC | left | HC vs PD-CN | Fusiform gyrus | 0.0610 | 1.09e-168 | 167.96 |
| EC | left | HC vs PD-CN | Posterior cingulate cortex | -0.0486 | 8.80e-131 | -130.06 |
| EC | left | HC vs PD-CN | Paracentral lobule | -0.0484 | 2.03e-101 | -100.69 |
| EC | left | HC vs PD-CN | Medial orbitofrontal cortex | -0.0329 | 4.20e-67 | -66.38 |
| EC | left | HC vs PD-CN | Isthmus of the cingulate gyrus | -0.0248 | 4.31e-34 | -33.37 |
| EC | left | HC vs PD-CN | Pars triangularis | -0.0252 | 1.72e-32 | -31.76 |
| EC | left | HC vs PD-CN | Lingual gyrus | -0.0223 | 4.59e-27 | -26.34 |
| EC | left | HC vs PD-CN | Frontal pole | 0.0227 | 3.20e-26 | 25.49 |
| EC | left | HC vs PD-CN | Parahippocampal gyrus | -0.0112 | 1.36e-25 | -24.87 |
| EC | left | HC vs PD-CN | Superior temporal gyrus | 0.0189 | 7.12e-25 | 24.15 |
| EC | left | HC vs PD-CN | Supramarginal gyrus | 0.0096 | 2.71e-20 | 19.57 |
| EC | left | HC vs PD-CN | Inferior parietal cortex | -0.0110 | 1.92e-19 | -18.72 |
| EC | left | HC vs PD-CN | Inferior temporal gyrus | -0.0156 | 4.55e-15 | -14.34 |
| EC | left | HC vs PD-CN | Caudal anterior cingulate cortex | -0.0046 | 2.89e-6 | -5.54 |
| EC | left | HC vs PD-CN | Pericalcarine cortex | -0.0078 | 5.11e-6 | -5.29 |
| EC | left | HC vs PD-CN | Rostral anterior cingulate cortex | -0.0057 | 1.08e-5 | -4.97 |
| EC | left | HC vs PD-CN | Entorhinal cortex | -0.0029 | 4.08e-5 | -4.39 |
| EC | left | HC vs PD-CN | Middle temporal gyrus | -0.0055 | 1.35e-4 | -3.87 |
| EC | left | HC vs PD-CN | Caudal middle frontal gyrus | 0.0057 | 0.003 | 2.58 |
| EC | left | HC vs PD-CN | Rostral middle frontal gyrus | 0.0041 | 0.007 | 2.18 |
| EC | left | HC vs PD-MCI | Medial orbitofrontal cortex | -0.0791 | < 1e-300 | < -300 |
| EC | left | HC vs PD-MCI | Parahippocampal gyrus | -0.0786 | < 1e-300 | < -300 |
| EC | left | HC vs PD-MCI | Superior parietal cortex | 0.1056 | < 1e-300 | > 300 |
| EC | left | HC vs PD-MCI | Fusiform gyrus | 0.0735 | 8.87e-312 | 311.05 |
| EC | left | HC vs PD-MCI | Rostral anterior cingulate cortex | -0.0659 | 5.35e-276 | -275.27 |
| EC | left | HC vs PD-MCI | Pars orbitalis | -0.0755 | 1.32e-253 | -252.88 |
| EC | left | HC vs PD-MCI | Superior frontal gyrus | 0.0507 | 2.96e-181 | 180.53 |
| EC | left | HC vs PD-MCI | Entorhinal cortex | -0.0263 | 1.21e-176 | -175.92 |
| EC | left | HC vs PD-MCI | Cuneus | -0.0594 | 2.31e-163 | -162.64 |
| EC | left | HC vs PD-MCI | Banks of the superior temporal sulcus | -0.0613 | 2.74e-157 | -156.56 |
| EC | left | HC vs PD-MCI | Insula | 0.0459 | 3.05e-147 | 146.52 |
| EC | left | HC vs PD-MCI | Lateral orbitofrontal cortex | -0.0555 | 6.15e-134 | -133.21 |
| EC | left | HC vs PD-MCI | Caudal anterior cingulate cortex | -0.0299 | 4.45e-128 | -127.35 |
| EC | left | HC vs PD-MCI | Pars triangularis | -0.0453 | 1.51e-94 | -93.82 |
| EC | left | HC vs PD-MCI | Lateral occipital cortex | -0.0483 | 5.55e-93 | -92.26 |
| EC | left | HC vs PD-MCI | Lingual gyrus | -0.0382 | 8.07e-92 | -91.09 |
| EC | left | HC vs PD-MCI | Precuneus | -0.0450 | 1.92e-75 | -74.72 |
| EC | left | HC vs PD-MCI | Inferior parietal cortex | 0.0351 | 1.44e-70 | 69.84 |
| EC | left | HC vs PD-MCI | Temporal pole | 0.0258 | 4.64e-60 | 59.33 |
| EC | left | HC vs PD-MCI | Transverse temporal gyrus | -0.0305 | 8.87e-56 | -55.05 |
| EC | left | HC vs PD-MCI | Postcentral gyrus | 0.0266 | 4.54e-53 | 52.34 |
| EC | left | HC vs PD-MCI | Rostral middle frontal gyrus | -0.0210 | 5.15e-51 | -50.29 |
| EC | left | HC vs PD-MCI | Paracentral lobule | -0.0278 | 3.83e-39 | -38.42 |
| EC | left | HC vs PD-MCI | Precentral gyrus | -0.0135 | 3.32e-9 | -8.48 |
| EC | left | HC vs PD-MCI | Pars opercularis | -0.0050 | 4.96e-8 | -7.30 |
| EC | left | HC vs PD-MCI | Inferior temporal gyrus | 0.0089 | 1.28e-6 | 5.89 |
| EC | left | PD-CN vs PD-MCI | Pars opercularis | -0.0771 | < 1e-300 | < -300 |
| EC | left | PD-CN vs PD-MCI | Parahippocampal gyrus | -0.0675 | 1.39e-276 | -275.86 |
| EC | left | PD-CN vs PD-MCI | Rostral anterior cingulate cortex | -0.0603 | 2.06e-225 | -224.69 |
| EC | left | PD-CN vs PD-MCI | Posterior cingulate cortex | 0.0506 | 5.58e-162 | 161.25 |
| EC | left | PD-CN vs PD-MCI | Banks of the superior temporal sulcus | 0.0564 | 5.49e-158 | 157.26 |
| EC | left | PD-CN vs PD-MCI | Entorhinal cortex | -0.0234 | 2.53e-137 | -136.60 |
| EC | left | PD-CN vs PD-MCI | Medial orbitofrontal cortex | -0.0462 | 2.07e-122 | -121.68 |
| EC | left | PD-CN vs PD-MCI | Lateral orbitofrontal cortex | 0.0462 | 2.55e-122 | 121.59 |
| EC | left | PD-CN vs PD-MCI | Inferior parietal cortex | 0.0460 | 3.60e-112 | 111.44 |
| EC | left | PD-CN vs PD-MCI | Postcentral gyrus | -0.0434 | 1.82e-111 | -110.74 |
| EC | left | PD-CN vs PD-MCI | Caudal anterior cingulate cortex | -0.0254 | 8.38e-92 | -91.08 |
| EC | left | PD-CN vs PD-MCI | Precuneus | 0.0406 | 4.91e-72 | 71.31 |
| EC | left | PD-CN vs PD-MCI | Rostral middle frontal gyrus | -0.0252 | 8.20e-63 | -62.09 |
| EC | left | PD-CN vs PD-MCI | Temporal pole | 0.0231 | 9.40e-51 | 50.03 |
| EC | left | PD-CN vs PD-MCI | Transverse temporal gyrus | 0.0227 | 1.54e-35 | 34.81 |
| EC | left | PD-CN vs PD-MCI | Superior parietal cortex | 0.0273 | 8.07e-33 | 32.09 |
| EC | left | PD-CN vs PD-MCI | Inferior temporal gyrus | 0.0245 | 7.04e-32 | 31.15 |
| EC | left | PD-CN vs PD-MCI | Isthmus of the cingulate gyrus | 0.0224 | 1.58e-31 | 30.80 |
| EC | left | PD-CN vs PD-MCI | Insula | -0.0224 | 2.12e-28 | -27.67 |
| EC | left | PD-CN vs PD-MCI | Frontal pole | -0.0185 | 6.47e-27 | -26.19 |
| EC | left | PD-CN vs PD-MCI | Pars triangularis | -0.0200 | 1.32e-22 | -21.88 |
| EC | left | PD-CN vs PD-MCI | Lateral occipital cortex | 0.0208 | 1.84e-22 | 21.74 |
| EC | left | PD-CN vs PD-MCI | Paracentral lobule | 0.0205 | 1.39e-18 | 17.86 |
| EC | left | PD-CN vs PD-MCI | Pars orbitalis | -0.0157 | 1.49e-17 | -16.83 |
| EC | left | PD-CN vs PD-MCI | Superior temporal gyrus | -0.0168 | 5.82e-17 | -16.23 |
| EC | left | PD-CN vs PD-MCI | Superior frontal gyrus | -0.0150 | 7.76e-14 | -13.11 |
| EC | left | PD-CN vs PD-MCI | Lingual gyrus | -0.0159 | 1.01e-13 | -12.99 |
| EC | left | PD-CN vs PD-MCI | Fusiform gyrus | 0.0125 | 3.87e-10 | 9.41 |
| EC | left | PD-CN vs PD-MCI | Precentral gyrus | -0.0130 | 1.56e-9 | -8.81 |
| EC | left | PD-CN vs PD-MCI | Pericalcarine cortex | 0.0065 | 3.42e-5 | 4.47 |
| EC | left | PD-CN vs PD-MCI | Supramarginal gyrus | -0.0069 | 4.62e-5 | -4.34 |
| EC | left | PD-CN vs PD-MCI | Cuneus | 0.0070 | 0.002 | 2.66 |
| EC | left | PD-CN vs PD-MCI | Caudal middle frontal gyrus | -0.0064 | 0.002 | -2.62 |
| EC | right | HC vs PD-CN | Banks of the superior temporal sulcus | -0.1015 | < 1e-300 | < -300 |
| EC | right | HC vs PD-CN | Lingual gyrus | -0.1024 | < 1e-300 | < -300 |
| EC | right | HC vs PD-CN | Paracentral lobule | -0.1098 | < 1e-300 | < -300 |
| EC | right | HC vs PD-CN | Pars opercularis | -0.0705 | < 1e-300 | < -300 |
| EC | right | HC vs PD-CN | Pars orbitalis | 0.1041 | < 1e-300 | > 300 |
| EC | right | HC vs PD-CN | Rostral anterior cingulate cortex | 0.0768 | < 1e-300 | > 300 |
| EC | right | HC vs PD-CN | Fusiform gyrus | 0.0799 | 1.26e-284 | 283.90 |
| EC | right | HC vs PD-CN | Medial orbitofrontal cortex | 0.0687 | 5.20e-230 | 229.28 |
| EC | right | HC vs PD-CN | Superior parietal cortex | 0.0583 | 2.64e-229 | 228.58 |
| EC | right | HC vs PD-CN | Lateral occipital cortex | -0.0646 | 5.64e-218 | -217.25 |
| EC | right | HC vs PD-CN | Isthmus of the cingulate gyrus | 0.0689 | 2.06e-216 | 215.69 |
| EC | right | HC vs PD-CN | Precuneus | -0.0764 | 3.11e-192 | -191.51 |
| EC | right | HC vs PD-CN | Posterior cingulate cortex | -0.0571 | 2.38e-177 | -176.62 |
| EC | right | HC vs PD-CN | Rostral middle frontal gyrus | 0.0380 | 1.55e-160 | 159.81 |
| EC | right | HC vs PD-CN | Transverse temporal gyrus | 0.0585 | 7.08e-160 | 159.15 |
| EC | right | HC vs PD-CN | Superior temporal gyrus | 0.0475 | 2.54e-136 | 135.59 |
| EC | right | HC vs PD-CN | Postcentral gyrus | 0.0405 | 6.57e-116 | 115.18 |
| EC | right | HC vs PD-CN | Precentral gyrus | 0.0422 | 1.14e-97 | 96.94 |
| EC | right | HC vs PD-CN | Superior frontal gyrus | 0.0180 | 1.84e-79 | 78.74 |
| EC | right | HC vs PD-CN | Insula | 0.0333 | 1.35e-67 | 66.87 |
| EC | right | HC vs PD-CN | Supramarginal gyrus | -0.0207 | 1.56e-60 | -59.81 |
| EC | right | HC vs PD-CN | Frontal pole | 0.0351 | 2.02e-53 | 52.70 |
| EC | right | HC vs PD-CN | Caudal middle frontal gyrus | -0.0156 | 2.44e-49 | -48.61 |
| EC | right | HC vs PD-CN | Entorhinal cortex | -0.0128 | 4.52e-28 | -27.34 |
| EC | right | HC vs PD-CN | Lateral orbitofrontal cortex | 0.0133 | 2.08e-14 | 13.68 |
| EC | right | HC vs PD-CN | Temporal pole | -0.0102 | 2.47e-11 | -10.61 |
| EC | right | HC vs PD-CN | Inferior parietal cortex | 0.0066 | 5.57e-9 | 8.25 |
| EC | right | HC vs PD-CN | Inferior temporal gyrus | -0.0106 | 2.78e-7 | -6.56 |
| EC | right | HC vs PD-CN | Parahippocampal gyrus | 0.0070 | 1.50e-6 | 5.82 |
| EC | right | HC vs PD-CN | Pericalcarine cortex | -0.0079 | 2.91e-6 | -5.54 |
| EC | right | HC vs PD-CN | Middle temporal gyrus | -0.0059 | 4.61e-5 | -4.34 |
| EC | right | HC vs PD-CN | Cuneus | 0.0067 | 4.14e-4 | 3.38 |
| EC | right | HC vs PD-CN | Pars triangularis | 0.0034 | 0.007 | 2.14 |
| EC | right | HC vs PD-CN | Caudal anterior cingulate cortex | -0.0035 | 0.009 | -2.03 |
| EC | right | HC vs PD-MCI | Entorhinal cortex | -0.0793 | < 1e-300 | < -300 |
| EC | right | HC vs PD-MCI | Inferior temporal gyrus | -0.1019 | < 1e-300 | < -300 |
| EC | right | HC vs PD-MCI | Superior frontal gyrus | 0.0766 | < 1e-300 | > 300 |
| EC | right | HC vs PD-MCI | Transverse temporal gyrus | 0.0720 | 4.99e-262 | 261.30 |
| EC | right | HC vs PD-MCI | Pars orbitalis | 0.0773 | 1.62e-251 | 250.79 |
| EC | right | HC vs PD-MCI | Superior parietal cortex | 0.0764 | 2.08e-226 | 225.68 |
| EC | right | HC vs PD-MCI | Parahippocampal gyrus | -0.0589 | 1.51e-216 | -215.82 |
| EC | right | HC vs PD-MCI | Postcentral gyrus | 0.0686 | 7.96e-215 | 214.10 |
| EC | right | HC vs PD-MCI | Pars opercularis | -0.0546 | 1.66e-184 | -183.78 |
| EC | right | HC vs PD-MCI | Fusiform gyrus | 0.0562 | 3.18e-177 | 176.50 |
| EC | right | HC vs PD-MCI | Insula | -0.0603 | 6.41e-177 | -176.19 |
| EC | right | HC vs PD-MCI | Caudal middle frontal gyrus | 0.0562 | 1.10e-156 | 155.96 |
| EC | right | HC vs PD-MCI | Paracentral lobule | -0.0470 | 3.11e-127 | -126.51 |
| EC | right | HC vs PD-MCI | Inferior parietal cortex | 0.0455 | 4.77e-109 | 108.32 |
| EC | right | HC vs PD-MCI | Posterior cingulate cortex | -0.0446 | 8.34e-109 | -108.08 |
| EC | right | HC vs PD-MCI | Lateral occipital cortex | -0.0497 | 2.36e-99 | -98.63 |
| EC | right | HC vs PD-MCI | Frontal pole | -0.0394 | 4.07e-70 | -69.39 |
| EC | right | HC vs PD-MCI | Precentral gyrus | 0.0399 | 2.81e-67 | 66.55 |
| EC | right | HC vs PD-MCI | Isthmus of the cingulate gyrus | 0.0377 | 8.41e-66 | 65.08 |
| EC | right | HC vs PD-MCI | Caudal anterior cingulate cortex | -0.0256 | 7.44e-57 | -56.13 |
| EC | right | HC vs PD-MCI | Pars triangularis | 0.0223 | 2.85e-53 | 52.55 |
| EC | right | HC vs PD-MCI | Temporal pole | -0.0226 | 9.43e-51 | -50.03 |
| EC | right | HC vs PD-MCI | Pericalcarine cortex | -0.0280 | 5.88e-44 | -43.23 |
| EC | right | HC vs PD-MCI | Superior temporal gyrus | 0.0220 | 8.51e-42 | 41.07 |
| EC | right | HC vs PD-MCI | Lateral orbitofrontal cortex | -0.0203 | 7.11e-32 | -31.15 |
| EC | right | HC vs PD-MCI | Rostral middle frontal gyrus | 0.0125 | 3.89e-24 | 23.41 |
| EC | right | HC vs PD-MCI | Cuneus | -0.0195 | 1.20e-19 | -18.92 |
| EC | right | HC vs PD-MCI | Lingual gyrus | 0.0124 | 6.22e-16 | 15.21 |
| EC | right | HC vs PD-MCI | Rostral anterior cingulate cortex | 0.0140 | 3.87e-11 | 10.41 |
| EC | right | HC vs PD-MCI | Supramarginal gyrus | 0.0087 | 2.22e-9 | 8.65 |
| EC | right | HC vs PD-MCI | Precuneus | -0.0119 | 3.72e-6 | -5.43 |
| EC | right | HC vs PD-MCI | Middle temporal gyrus | -0.0049 | 0.005 | -2.35 |
| EC | right | HC vs PD-MCI | Medial orbitofrontal cortex | -0.0047 | 0.028 | -1.55 |
| EC | right | PD-CN vs PD-MCI | Banks of the superior temporal sulcus | 0.0988 | < 1e-300 | > 300 |
| EC | right | PD-CN vs PD-MCI | Lingual gyrus | 0.1148 | < 1e-300 | > 300 |
| EC | right | PD-CN vs PD-MCI | Insula | -0.0937 | 2.45e-309 | -308.61 |
| EC | right | PD-CN vs PD-MCI | Inferior temporal gyrus | -0.0913 | 8.34e-286 | -285.08 |
| EC | right | PD-CN vs PD-MCI | Frontal pole | -0.0745 | 8.17e-271 | -270.09 |
| EC | right | PD-CN vs PD-MCI | Parahippocampal gyrus | -0.0659 | 5.80e-247 | -246.24 |
| EC | right | PD-CN vs PD-MCI | Superior frontal gyrus | 0.0585 | 1.79e-245 | 244.75 |
| EC | right | PD-CN vs PD-MCI | Entorhinal cortex | -0.0665 | 2.91e-244 | -243.54 |
| EC | right | PD-CN vs PD-MCI | Medial orbitofrontal cortex | -0.0734 | 1.04e-225 | -224.98 |
| EC | right | PD-CN vs PD-MCI | Caudal middle frontal gyrus | 0.0718 | 1.25e-220 | 219.90 |
| EC | right | PD-CN vs PD-MCI | Paracentral lobule | 0.0628 | 1.94e-168 | 167.71 |
| EC | right | PD-CN vs PD-MCI | Precuneus | 0.0646 | 1.30e-165 | 164.89 |
| EC | right | PD-CN vs PD-MCI | Rostral anterior cingulate cortex | -0.0628 | 3.29e-164 | -163.48 |
| EC | right | PD-CN vs PD-MCI | Isthmus of the cingulate gyrus | -0.0312 | 3.20e-101 | -100.50 |
| EC | right | PD-CN vs PD-MCI | Inferior parietal cortex | 0.0389 | 5.69e-83 | 82.24 |
| EC | right | PD-CN vs PD-MCI | Lateral orbitofrontal cortex | -0.0335 | 2.76e-74 | -73.56 |
| EC | right | PD-CN vs PD-MCI | Supramarginal gyrus | 0.0294 | 7.62e-69 | 68.12 |
| EC | right | PD-CN vs PD-MCI | Rostral middle frontal gyrus | -0.0255 | 5.47e-55 | -54.26 |
| EC | right | PD-CN vs PD-MCI | Caudal anterior cingulate cortex | -0.0221 | 2.18e-52 | -51.66 |
| EC | right | PD-CN vs PD-MCI | Fusiform gyrus | -0.0238 | 9.59e-42 | -41.02 |
| EC | right | PD-CN vs PD-MCI | Postcentral gyrus | 0.0281 | 6.09e-35 | 34.22 |
| EC | right | PD-CN vs PD-MCI | Superior temporal gyrus | -0.0255 | 2.43e-33 | -32.61 |
| EC | right | PD-CN vs PD-MCI | Pars orbitalis | -0.0269 | 2.98e-33 | -32.53 |
| EC | right | PD-CN vs PD-MCI | Pars triangularis | 0.0189 | 1.31e-29 | 28.88 |
| EC | right | PD-CN vs PD-MCI | Pericalcarine cortex | -0.0201 | 6.53e-28 | -27.18 |
| EC | right | PD-CN vs PD-MCI | Cuneus | -0.0262 | 1.55e-26 | -25.81 |
| EC | right | PD-CN vs PD-MCI | Pars opercularis | 0.0159 | 8.60e-19 | 18.07 |
| EC | right | PD-CN vs PD-MCI | Temporal pole | -0.0124 | 2.62e-17 | -16.58 |
| EC | right | PD-CN vs PD-MCI | Superior parietal cortex | 0.0181 | 1.04e-15 | 14.98 |
| EC | right | PD-CN vs PD-MCI | Transverse temporal gyrus | 0.0136 | 5.79e-15 | 14.24 |
| EC | right | PD-CN vs PD-MCI | Lateral occipital cortex | 0.0149 | 1.36e-10 | 9.87 |
| EC | right | PD-CN vs PD-MCI | Posterior cingulate cortex | 0.0125 | 1.52e-9 | 8.82 |
